# Bridge helix bending promotes RNA polymerase II backtracking through a critical and conserved threonine residue

**DOI:** 10.1038/ncomms11244

**Published:** 2016-04-19

**Authors:** Lin-Tai Da, Fátima Pardo-Avila, Liang Xu, Daniel-Adriano Silva, Lu Zhang, Xin Gao, Dong Wang, Xuhui Huang

**Affiliations:** 1 Department of Chemistry, School of Science and Institute for Advance Study, Hong Kong University of Science and Technology, Clear Water Bay, Kowloon, Hong Kong; 2 Department of Cellular and Molecular Medicine, School of Medicine; Skaggs School of Pharmacy and Pharmaceutical Sciences, University of California San Diego, La Jolla, California 92093, USA; 3 Department of Biochemistry, University of Washington, Seattle, Washington 98195, USA; 4 King Abdullah University of Science and Technology, Computational Bioscience Research Center, Computer, Electrical and Mathematical Sciences and Engineering Division, Thuwal 23955-6900, Saudi Arabia; 5 Division of Biomedical Engineering, School of Science and Institute for Advance Study, Hong Kong University of Science and Technology, Clear Water Bay, Kowloon, Hong Kong; 6 Center of Systems Biology and Human Health, School of Science and Institute for Advance Study, Hong Kong University of Science and Technology, Clear Water Bay, Kowloon, Hong Kong

## Abstract

The dynamics of the RNA polymerase II (Pol II) backtracking process is poorly understood. We built a Markov State Model from extensive molecular dynamics simulations to identify metastable intermediate states and the dynamics of backtracking at atomistic detail. Our results reveal that Pol II backtracking occurs in a stepwise mode where two intermediate states are involved. We find that the continuous bending motion of the Bridge helix (BH) serves as a critical checkpoint, using the highly conserved BH residue T831 as a sensing probe for the 3′-terminal base paring of RNA:DNA hybrid. If the base pair is mismatched, BH bending can promote the RNA 3′-end nucleotide into a frayed state that further leads to the backtracked state. These computational observations are validated by site-directed mutagenesis and transcript cleavage assays, and provide insights into the key factors that regulate the preferences of the backward translocation.

## Abstract (web summary)

RNA Polymerase II can detect and cleave mis-incorporated nucleotides by a proofreading mechanism that requires backtracking of the enzyme. Here the authors show that Pol II backtracking occurs in a stepwise mode that involves two intermediate states where the fraying of the terminal RNA nucleotide is a prerequisite.

## Graphical abstract

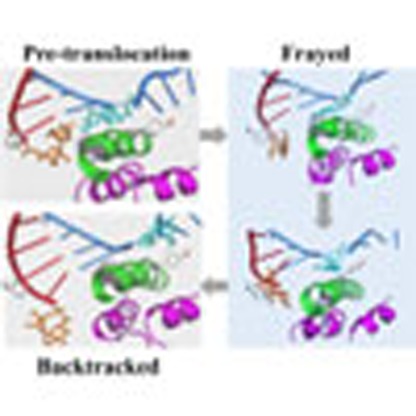

RNA polymerase is the key enzyme in gene expression responsible for synthesizing messenger RNA (mRNA) based on the DNA template^1^,^2^,^3^,^4^. RNA polymerase II (Pol II) can efficiently detect and cleave mis-incorporated nucleotides via its proofreading mechanism that contributes its high transcription fidelity^5^,^6^. The transcription proofreading is enabled by Pol II backtracking (see Fig. 1a for details): Pol II moves in a reverse direction from the pre-translocation state to a ‘backtracked' state where the RNA 3′-end nucleotide dislodges from the active site, and extrudes through the pore region of the secondary channel^7^,^8^. This backtracking motion can be greatly favoured by damaged DNA template, mismatched base-pairing or a nucleosomal barrier^9^,^10^,^11^,^12^ (see Fig. 1b for details). Finally, 1 or 2 backtracked RNA nucleotides can be removed via an intrinsic activity of Pol II, whereas the cleavage of more than two backtracked RNA nucleotides requires the recruitment of the transcription factor IIS (TFIIS). The backtracked Pol II Elongation Complex (EC) has been implicated in many critical biological processes, such as control of promoter-proximal pausing, transcription elongation dynamics (pausing and arrest), termination and so on (refs 10, 13, 14, 15, 16).

Recently, the X-ray structures of Pol II EC arrested in backtracked states have been solved^7^,^8^. Wang *et al*.^8^ revealed atomistic details of a backtracked Pol II EC with one or two RNA 3′-end nucleotides retreated to the backtracked form. This allows identifying the critical interactions between the backtracked RNA nucleotides and the Pol II residues. In particular, a single backtracked RNA 3′-end nucleotide was found to directly contact the Rpb2 residue Y769, Trigger Loop (TL) residues Q1078, N1082 and Bridge helix (BH) residues T827 and so on. More recently, Cheung *et al*.^7^ employed CTP extension from a tailed DNA template and captured the X-ray structure of the Pol II EC with nine backtracked poly(C) RNA transcript, providing the details of the extended interaction network between the further backtracked RNA nucleotides and the Pol II residues. In addition, Sydow *et al*.^17^ reported a structure of Pol II EC in a frayed state (not yet backtracked), in which the RNA 3′-end frayed nucleobase stacks directly with the Rpb2 residue Y769, this structure was thus suggested as an intermediate state before the complete backtracking^17^ (see the fraying-dependent stepwise model in Supplementary Fig. 1). However, the frayed state was not crystallized with a 3′-end mismatched ribonucleotide in the active site, instead, the mismatched nucleotides were located at an upstream position. Therefore, there still exists the possibility that this frayed state is an off-pathway metastable state, and 3′-end RNA nucleotide can backtrack concurrently with backtracking of its complementary template DNA nucleotide (see the fraying-independent concerted model in Supplementary Fig. 1). Although the above X-ray structures have revolutionized our understandings of the backtracked Pol II ECs at an atomic detail, several critical questions remain largely unsolved. For example, Does backtracking take place in a stepwise or a concerted mode? (that is, Whether the terminal base pair is disrupted before or co-currently in comparison with pol II reverse translocation?) What is the driving force for the backtracking? How does Pol II modulate the energetics of backtracking when mismatch or DNA damage is present?

The experimental approaches to address the above issues are limited and challenging, as most of these approaches cannot directly provide dynamical information for backtracking at the molecular level. Molecular dynamics (MD) simulations can greatly complement experimental observations on elucidating the dynamics at the atomic level. MD simulations have been widely applied to study proteins and nucleic acids, including systems as large as Pol II (refs 18, 19, 20, 21, 22, 23, 24). One major challenge faced by MD simulations of a system as large as the Pol II complex in an explicit solvent box (∼370,000 atoms) is to reach biologically relevant timescales^25^. Most all-atom MD simulations of RNA polymerases are performed at timescales from tens to hundreds of nanoseconds, whereas relevant conformational changes such as backtracking typically occur at timescales on the order of hundreds of microseconds or even longer^26^.

The above-mentioned timescale gap can be bridged by Markov State Models (MSMs), which allow us to model long timescale dynamics from many parallel short MD simulations^27^,^28^,^29^,^30^,^31^,^32^,^33^,^34^,^35^,^36^,^37^. MSMs have been successfully employed to investigate conformational changes that are difficult to be studied using straightforward MD simulations^37^,^38^. These studies cover a wide aspect of areas, including protein^39^,^40^,^41^,^42^,^43^ or RNA^32^ conformational changes, ligand-receptor binding^44^,^45^,^46^, allostery^47^ and so on. Recently, we have constructed a MSM to elucidate the forward translocation mechanism of Pol II EC at atomic resolution, and found that the translocation in Pol II EC takes place at the timescale of tens of microseconds in the absence of incoming nucleoside triphosphate (NTP)^48^.

In this study, we built a MSM to investigate the mechanisms of Pol II backtracking upon nucleotide mis-incorporation, a critical step for proofreading. We identified a stepwise backtracking process in which the first step is the fast fraying motion (at sub-microsecond) of the RNA 3′-end nucleotide. This step is followed by the rotation of the DNA transition nucleotide (TN) to stack with BH residues, which results in the formation of an additional intermediate state. During the last step, the reverse translocation of the upstream RNA:DNA hybrid takes place at around 100 μs to reach the final backtracked register. Interestingly, we found that the bending of the BH can serve as a checkpoint that examines the stability of the base pair (bp) of RNA:DNA hybrid at *i*+1 site, and determine the direction of translocation (whether the RNA:DNA hybrid moves forward or backward by fraying and backtracking). In particular, the conserved BH residue T831, which directly points at the *i*+1 site, can facilitate the separation of the mismatched base pair and drive the Pol II complex into the backtracked state. The critical role of T831 predicted by simulations was further validated by site-directed mutagenesis experiments, which show that the T831A substitution can substantially decrease the backtracking-dependent transcript cleavage rate.

## Results

### Backtracking in RNA Pol II EC follows a stepwise model

We first set up the frayed and one-nucleotide backtracked states based on the crystal structures with PDB ID: 3HOZ^17^ and 3GTG^8^, respectively (Supplementary Fig. 1). The pre-translocation state was modelled by placing an rG:dG mismatched base pair in the active site and removing the backtracked nucleotides from the crystal structure (PDB ID: 3GTG). Next, we obtained the initial backtracking pathways using the Climber algorithm^49^, which is designed to search for the low-energy pathways connecting two conformational states. To take into account the possibility of both stepwise and concerted mechanisms, we generated initial pathways following both models, that is, pre-translocation ↔ backtracked and pre-translocation ↔ frayed ↔ backtracked states. Finally, to eliminate the bias introduced by the initial pathways, three rounds of MD simulations were performed and only the last two rounds of MD simulations (with an aggregated simulation time of ∼48 μs) were used to construct the final MSM (see the Methods section for details).

**Figure 2:**
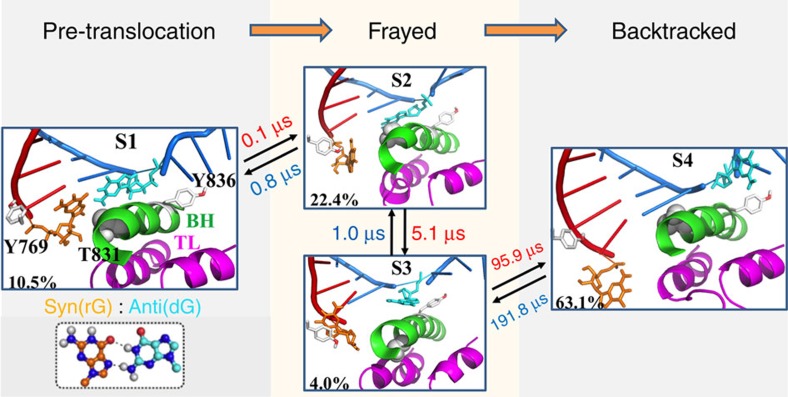
Backtracking in RNA Pol II EC follows a stepwise model. Representative structures for each of the four metastable states identified by our Markov State Model (MSM; S1–S4) and connected by the top one pathway from the pre-translocation state (S1) to the backtracked state (S4): S1→S2→S3→S4. The equilibrium population for each state (left bottom corner) and the Mean First Passage Time (MFPT) between each transition (beside arrow, unit in μs) are presented, with their corresponding errors given as followings: 10.5±1.5%, 22.4±2.1%, 4.0±0.7% and 63.1±2.8% (equilibrium population); 0.1±0.0 μs, 0.8±0.1 μs, 5.1±3.0 μs, 1.0±0.7 μs, 95.9±42.3 μs and 191.8±69.0 μs (MFPT). The hybrid RNA/DNA chains (red/blue), the Trigger Loop (purple), Bridge helix (green), Rpb1 residue Y836 and Rpb2 residue Y769 (both in grey) are shown. The DNA TN (dG) and its mismatched RNA nucleotide (rG) are highlighted with cyan and orange stick model, respectively.

From the MSM, we found that backtracking follows a stepwise mechanism rather than a concerted one. In particular, we used Transition Path Theory to compute the probability of each of the two competing mechanisms of backtracking from the flux through each pathway^41^,^50^. This flux analysis shows that the entire flux goes through the stepwise pathway (see Fig. 2 and Supplementary Movie 1 for details). The projection of the free energy landscapes on a pair of reaction coordinates (root-mean-square deviations (RMSDs) of the two RNA 3′-end nucleotides and their corresponding base-paired DNA nucleotides) obtained from our MSM also clearly shows that the RNA fraying and DNA template translocation occur in a stepwise mode (see Supplementary Fig. 2a for details). Furthermore, we observe that one of the initial pathways corresponding to the concerted mechanism (the black line in Supplementary Fig. 2a) clearly deviates from the low free energy regions of the MSM projection (contour lines in Supplementary Fig. 2a). To further prove that the MSM is capable of eliminating the bias introduced by the initial pathways, we projected the conformations from our MSM sampling onto three reaction coordinates (three top eigenvectors identified by the Isomap dimensionality reduction technique)^51^. As shown in Supplementary Fig. 2b, the MSM sampling largely overlaps with the stepwise initial pathways (in yellow), even though it covers a much larger region of the conformational space. However, the MSM sampling clearly deviates from the concerted initial pathways (in cyan). In summary, our MSM built upon the wild-type (WT) MD simulations has clearly indicated the existence of the frayed intermediate state and supported the stepwise model, whereas strongly disfavouring the concerted mechanism.

Our MSM for backtracking contains four metastable states (S1–S4 states; Fig. 2). As observed in the crystal structure^5^, favourable interactions between the RNA 3′-end nucleotide and a number of Pol II residues (for example, BH residues T827, and TL residues Q1078 and N1082) are formed to stabilize the backtracked state (S4) for the rG:dG mismatched system (see Supplementary Fig. 1 for details). In addition to the pre-translocation (S1) and backtracked states (S4), two additional intermediate states are identified along the major pathway (Fig. 2). One of them (S2) is consistent with the frayed crystal structure^17^. The other intermediate also contains a frayed RNA 3′-end nucleotide, but two additional interactions are formed: the BH residue Y836 interacts with the DNA TN, and Rpb2 residue Y769 stacks with the RNA 3′-end nucleotide (Fig. 2). These two residues play a critical role in stabilizing the frayed RNA nucleotide in the S3 state. In particular, the stable stacking interactions between the base group of the frayed RNA nucleotide and the aromatic ring of the Y769 exist in most of the MD conformations (∼80%) from the S3 state. However, only limited conformations (<10%) from the other three states contain the above stacking interactions. The transition between the intermediate state S3 and the backtracked state (S4) that requires the translocations of the upstream RNA:DNA hybrid is the rate-limiting step (Fig. 2), at an order of a 100 μs. In addition, the system can quickly inter-convert between the other three states (S1–S3) at a timescale of a few microseconds. Finally, the backtracked state is the most stable state (∼63.1% of the conformations), whereas the pre-translocation state is much less stable with a population around an order of magnitude smaller, consistent with the kinetic model derived from a recent single-molecule experimental study^12^. These results strongly suggest that the rG:dG mismatched base pair can significantly shift the translocation bias towards the backtracked state.

### RNA 3′-end nucleotide frays due to mismatch and BH bending

**Figure 3:**
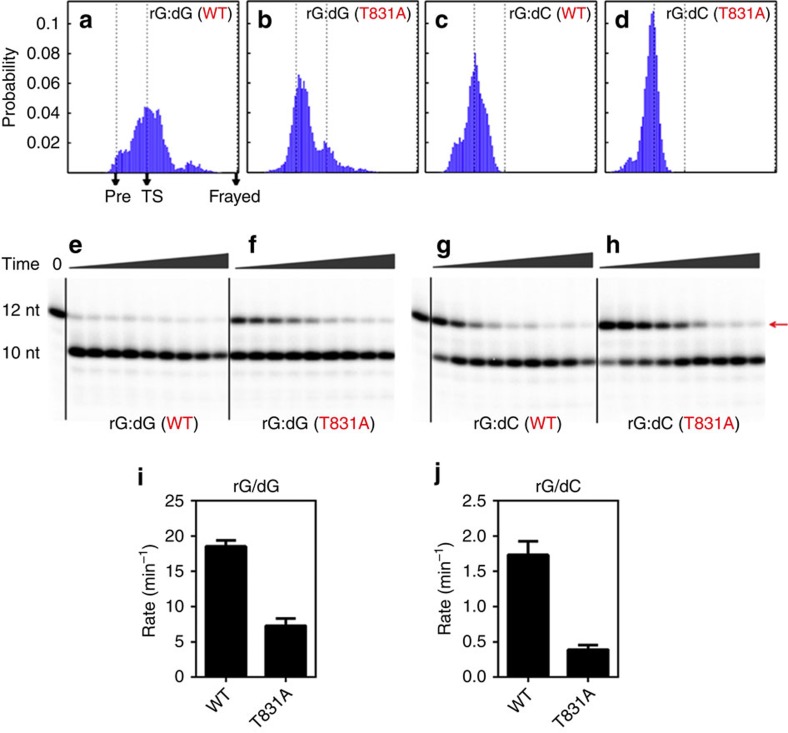
Fraying motion of the RNA 3′-end nucleotide is dictated by the mismatched base pair in the active site and promoted by BH residue T831 serving as a sensing probe. As a comparison, one additional matched base pair (rG:dC) was designed for the mutant MD simulations starting from the regions near to the transition state (TS) between S1 and S2 states. (**a**–**d**) Histogram for the movements of the RNA 3′-end nucleotide for the rG:dG (**a**) and rG:dC (**c**) base pairs starting from the above TS, and their corresponding T831A mutants on the Pol II BH domain (**b** and **d**, respectively). Three critical states: Pre-translocation state (S1), TS and Frayed state (S2), are mapped with dashed lines and black arrows in the plots. The *x* coordinate represents a transition index, defined as 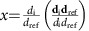, where **d**_i_=**r**_MD_−**r**_pre_ is the vector connecting the pre-translocation structure and a certain MD conformation (each conformation **r** is represented using the c.o.m coordinates of its DNA TN). **d**_ref_=**r**_frayed_−**r**_pre_ is the reference vector connecting the pre-translocation and frayed state. The transition index indicates where a MD conformation locates between the pre-translocation and frayed state. In particular, *x*=0 corresponds to the pre-translocation (pre) state while *x*=1 corresponds to the frayed state. (**e**–**h**) Evaluation of the functional roles of the BH residue T831 in backtracking by site-directed mutagenesis studies and TFIIS cleavage assays. Comparing to the WTs of the rG:dG and rG:dC systems (**e** and **g**, respectively), the corresponding T831A mutant has weaker TFIIS-facilitated cleavage activities (**f** and **h**, respectively). The upper bands refer to the initial RNA transcript (12 nt) as indicated by red arrow; the lower bands refer to the cleaved product (10 nt). Quantitative analysis of TFIIS-stimulated cleavage rates is shown in **i** and **j**. Error bars represent standard deviations derived from three independent experiments.

As shown in Fig. 2, for the rG:dG mismatched base pair, the frayed state (S2, 22.4%) is energetically more favourable than the pre-translocation state (S1, 10.5%). To evaluate the effects of the fully matched base pair on the stability of the frayed state, we designed an additional Pol II EC with rG:dC bp at *i*+1 site for further MD simulation studies (Fig. 3). To obtain the starting structures, we chose seven representative conformations near the transition state (TS) region that separates the S1 and S2 metastable states of the rG:dG system (corresponding to the region around the RMSD coordinate (3, 2.5) in the free energy profile in Supplementary Fig. 2a), and then mutated the rG:dG base pair to the rG:dC bp. Next, we performed 10-ns MD simulations for each of the above newly generated conformations with both rG:dC and rG:dG base pair systems.

The results clearly show that for the rG:dG base pair, nearly half of the conformations (46%) move back to the S1 state (Fig. 3a), indicating that the selected conformations indeed locate near the TS between the S1 and S2 states involved in the backtracking process for the rG:dG system. In sharp contrast, the rG:dC matched base pair completely shifts the transition equilibrium towards the S1 state (∼100%; Fig. 3c), suggesting that the hydrogen bonds of the rG:dC base pair significantly favour the pre-translocation state and prevent the RNA 3′-end nucleotide from fraying. Our mutant MD simulation results strongly support the idea that the strength of the hydrogen bonds within the base pair located in the active site can significantly modulate the relative stabilities of S1 and S2 states, which in turn can shift the backtracking bias. Consistently, the experiments conducted by Wang and co-workers showed that the RNA cleavage efficiencies for the rG:dG pair was significantly higher than that for the rG:dC base pair^8^. In addition to the rG:dG mismatch, we also performed MD simulations from the TS between S1 and S2 for three other mismatched base pairs in the active site: rA:dG, rC:dT and rU:dT. For all these three systems, we also observed a significant fraction of MD simulations that transit towards the frayed state (S2), which indicates that the frayed state could be a metastable intermediate for different mismatched systems (see Supplementary Fig. 3 and Supplementary Note 6 for details).

**Figure 4:**
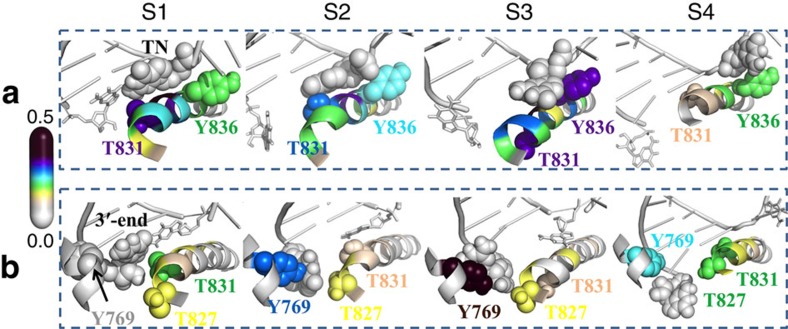
Functional roles of the Pol II BH residues during the backtracking revealed by cross-correlation analysis. In each metastable state, the correlations were calculated between the Pol II residues and the DNA TN (**a**) or RNA 3′-end nucleotide (**b**), respectively. For DNA TN (**a**), two critical BH residues, T831 and Y836, are highlighted with sphere models. In both S1 and S2 states, residue T831 is tightly correlated with the TN. Compared with S1 state, the DNA TN enhances its correlations with the residue Y836 in S2 state because of increased flexibility. In S3 state, the TN further increases its correlations with residue Y836 by forming direct packing interactions, which is critical for the TN to cross over the BH. In S4 state, the tight correlation between the Y836 and TN remains. For the RNA 3′-end nucleotide (**b**), two BH residues, T827 and T831, and one Rpb2 residue Y769, are highlighted as sphere models. In S1 state, the T831 shows the strongest correlation with the RNA 3′-end nucleotide, and weakly correlated with T827 and Y769. In S2 state, the RNA 3′-end nucleotide loses the coupling with residue T831 and strengthens the correlations with residues T827 and Y769. Strikingly, in S3 state, the RNA 3′-end nucleotide is tightly coupled to the residue Y769 by forming stable packing interactions, and it is barely coupled with BH residues. In the final state, RNA 3′-end nucleotide shows the correlation with all of these three residues. For each representation, the RNA:DNA hybrid chains are shown in grey, the DNA TN in **a** and RNA 3′-end nucleotide in **b** are shown as grey sphere models. The colours of the Pol II residues, including the BH residues and the Rpb2 residue Y769, correspond to the colours in the spectrum bar on the left.

To reveal the functional roles of the BH in the fraying motion of the RNA 3′-end nucleotide, we carried out a cross-correlation analysis for both the RNA 3′-end nucleotide and the DNA TN (Fig. 4). We found that in the S1 state, the BH residue T831 is tightly coupled with both RNA and DNA nucleotides in the active site (Fig. 4a,b). Interestingly, the residue T831 was found to locate right on the helical turn of the BH that bends the most (Supplementary Fig. 4). Therefore, we suggest that the BH serves as a checkpoint to examine the stability of the base pair in the active site through its bending motion, using residue T831 as a sensing probe. In other words, if the base pair in the active site is unstable (for example, mismatched), the RNA 3′-end nucleotide tends to fray, which can be further promoted by the continuous bending motion of the BH. Next, in the frayed state, the motion of the RNA 3′-end nucleotide becomes less correlated with that of T831, whereas the motion of RNA 3′-end nucleotide becomes more correlated with that of the BH residue T827 and Rpb2 residue Y769 (Fig. 4a,b). On the other hand, the dynamics of the DNA TN is still highly correlated with T831, and establishes new correlations with the dynamics of the BH residue Y836 (Fig. 4a). This may be due to the increased flexibility of the DNA TN after breaking its interactions with the RNA 3′-end nucleotide.

### Site-directed mutagenesis validates role of T831

To further examine the specific roles of the BH residue T831 in backtracking, we first performed additional MD simulations for the rG:dG system with the T831A mutation on the BH. We started the simulations from the same set of conformations near the TS between S1 and S2 states, as used in the above MD simulations of different base pairs at the *i*+1 site. The results indicate a significant difference between the WT and the T831A mutant. As shown in Fig. 3b, the T831A substitution on the BH can greatly prevent the fraying of the RNA 3′-end nucleotide with nearly 86% conformations moving towards the S1 state, sharply contrasting to the corresponding WT results (Fig. 3a), suggesting that the BH residue T831 can substantially facilitate the fraying motion of the RNA 3′-end nucleotide.

To validate the above prediction, we conducted mutagenesis studies to evaluate the role of the BH residue T831 by measuring the transcript cleavage rate, which is dependent on the extent of the backtracking. Based on our model, the T831A substitution on Pol II can diminish the ability of the BH domain to promote the fraying of the RNA 3′-end nucleotide, which, in turn, would slow down the cleavage reaction. We designed the same RNA:DNA scaffold for the rG:dG system as we used in our theoretical studies and measured the cleavage rate with both WT and T831A mutant of Pol II EC. Here, we employed both intrinsic and TFIIS-stimulated transcript cleavage assays to probe the impact of T831A on backtracking behaviour of RNA pol II. Indeed, as expected, we found reduced transcript cleavage activity with T831A pol II mutant in comparison with WT pol II (see Fig. 3e,f and Supplementary Fig. 5 for details). Intriguingly, we observed much more striking reduction of cleavage activities for T831A pol II mutant in the TFIIS-mediated cleavage. Quantitative analysis indicates the substitution T831A can significantly reduce the TFIIS-aided transcript cleavage rate by 2.5-fold compared with the WT (Fig. 3i), which provides experimental verification for our theoretical prediction.

As a control, we further evaluated the effects of the T831A mutation on the rG:dC system by performing both MD simulations and experimental studies, following the same procedure as we did for the rG:dG system. The MD simulations show that, for the rG:dC system, the T831A substitution slightly favours the forward translocation over the fraying motions (the population ratio between the conformations that translocate forward and the conformations that tend to fray is ∼69% versus 31%; Fig. 3d), whereas the above distribution is reversed in the WT where the population ratio is 46% versus 54% (see Fig. 3c). Consistent with the above theoretical observation, the intrinsic and TFIIS-stimulated cleavage experiments (Fig. 3g,h, j and Supplementary Fig. 5) also support that, for the rG:dC system, the presence of T831 in WT could substantially promote the backtracking process and thus increase cleavage rates (Fig. 3g), compared with the T831A mutant (Fig. 3h). Notably, we found the effect of substitution of T831A on TFIIS-stimulated cleavage (experiment data) is much more significant than intrinsic cleavage (both experiment and simulation). This result suggests that a tighter coupling of tri-helical bundle (composed of BH and helical portions of TL) interaction upon TFIIS insertion within the secondary channel, thus the BH bending and T831 may have much more significant effect on backtracked translocation.

### Backtracking of the upstream RNA:DNA hybrid

**Figure 5:**
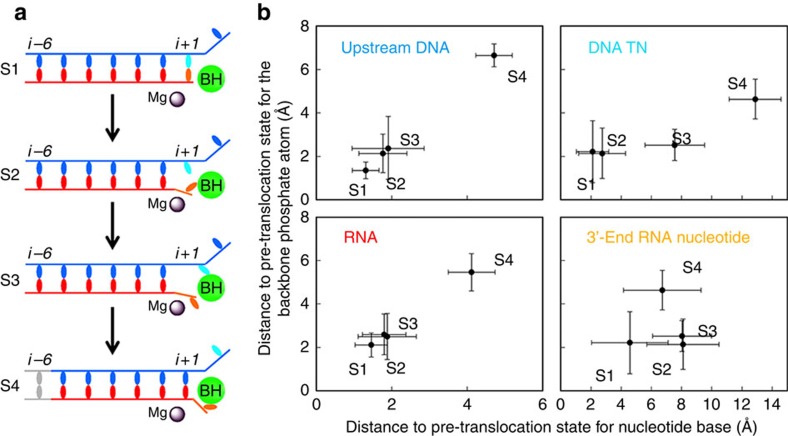
Fraying of the RNA 3′-end nucleotide and the entry of the DNA TN into the backtracking pathway are not accompanied by translocation of the RNA:DNA hybrid. (**a**) Cartoon models illustrating the movements of the RNA 3′-end nucleotide (orange), DNA TN (cyan) and upstream RNA:DNA hybrids (red/blue) during the backtracking. From S1 to S2, the RNA 3′-end nucleotide frays from the *i*+1 site, but the DNA TN remains at *i*+1 site. From S2 to S3, the DNA TN starts to cross over the BH and stacks with the BH residues. From S3 to S4, the backbone translocation of the upstream RNA:DNA hybrids occurs at a single-step and facilitates the DNA TN to transfer to the complete backtracked state. (**b**) Plots showing the distance to the pre-translocation state for the backbone phosphate and the nucleotide base. For the upstream DNA chain (upper left panel) and RNA chain (lower left panel), the distance values were averaged over 5 upstream DNA and RNA nucleotides (From *i*-2 to *i*-6 sites, see Supplementary Fig. 3). The panels on the right show only the RNA 3′-end nucleotide and DNA TN (lower and upper rows, respectively). The fluctuations of the distance within each state are indicated with the error bars.

We found that the fraying of the RNA 3′ nucleotide and the entry of the DNA TN into the backtracking pathway are not accompanied by translocation of the RNA:DNA hybrid. As shown in Fig. 5 and Supplementary Fig. 6, the upstream RNA:DNA hybrid backtracks in a single step during the S3 to S4 transition, and this motion occurs after the fraying of the RNA 3′-end nucleotide and the formation of the stable base stacking between DNA TN and BH residue Y836 (Fig. 4). In our model, the backtracking of the upstream RNA:DNA hybrid is the rate-limiting step of the whole backtracking process (at ∼100 μs), as it requires the breaking and re-forming of numerous interactions between Pol II and the RNA:DNA hybrid.

This asynchronous movement of the hybrid backbones and DNA TN is also observed in the forward translocation process for the matched nucleotide system^48^. In the absence of incoming NTP, the Pol II EC with matched nucleotides can oscillate between the pre- and post-translocation state. For the forward translocation, the upstream RNA:DNA hybrid movement is also the rate-limiting step, but its timescale is ∼5-fold faster than what we observed for the backtracking. The slower dynamics of backtracking is likely due to the breaking of the stacking interactions between the frayed RNA nucleotide and the Rpb2 residue Y769 during the transition from S3 to S4, as the Y769 is tightly correlated and forms direct contacts with the frayed RNA 3′-end nucleotide in the S3 state, but the correlation is largely lost in the S4 state (Fig. 4). In addition, the aromatic side chain of the BH residue Y836 is found to form direct stacking interactions with the base of DNA TN in S3 (Figs 2 and 4).

To reveal the specific role of the above two residues Y836 and Y769 in the transition of the DNA TN from S3 to S4 states, we chose four representative conformations near the TS between the states S3 and S4 (the region with the RMSD value of ∼6.3 Å along the *Y* axis in the free energy profile in Supplementary Fig. 2a). For those four selected conformations, we then replaced the Y836 and Y769 individually with alanine, and performed 10 ns MD simulations for each of the mutant systems as well as the WT (see the Methods section for details). Our results show that the Y836A mutation can prevent the transition of the DNA TN to the S4 state (∼14% MD conformations move towards S4 state) compared with the WT system (Supplementary Fig. 7a,b). In a previous forward translocation study^48^, Y836 has been suggested to stabilize the DNA TN over the BH and further facilitate the translocation of the upstream RNA:DNA hybrid. We thus suggest that Y836 plays a similar role in facilitating the transition from the S3 to S4 state during the backtracking. However, the substitution Y769A can conversely promote the backtracking with ∼71% MD conformations moving towards S4 state (Supplementary Fig. 7c), supporting our previous conclusion that Y769 can substantially prevent the backtracking process.

## Discussion

**Figure 6:**
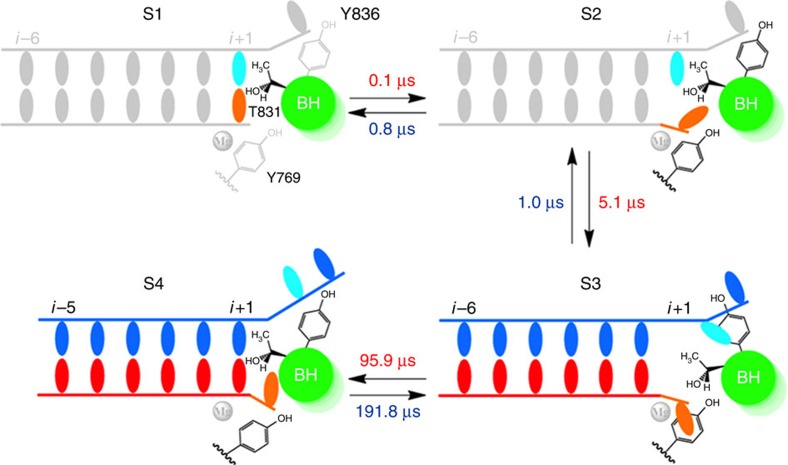
Cartoon model of the backtracking mechanism in Pol II EC. Bending of the BH towards the *i*+1 site serves as a checkpoint to examine the stabilities of the base pair in the active site using residue T831 as a sensing probe. If the base pairing at *i*+1 is unstable (for example, mismatched), BH bending can trigger the motion of the RNA 3′-end nucleotide to the frayed state (S2 state). Next, the Pol II EC moves to S3 state where BH residue Y836 stacks with DNA TN and Rpb2 residue Y769 stacks with RNA 3′-end nucleotide through their aromatic rings. Finally, the movement of the RNA:DNA hybrid takes place. Refer to the Fig. 4a for details of the representations. The MFPTs for each transition calculated from the MSM are also provided.

We revealed atomic-level details of the molecular mechanism of the backtracking in Pol II EC by constructing a MSM from extensive MD simulations, combined with site-directed mutagenesis and transcript cleavage studies. We employed the rG:dG mismatched base pair in our study and found that the backtracking occurred in a stepwise mode comprising four well-defined metastable states. At the beginning, the pre-translocation state (S1) can quickly move to the frayed state (S2), which is promoted by the continuous bending motions of BH (Fig. 6). We suggest that the bending motion of the BH works as a checkpoint that can examine the stability of the base pair in the active site using the residue T831 as a probe that interacts with the base groups of both DNA TN and RNA 3′-end nucleotide (Fig. 6), which is well consistent with our mutagenesis results. Next, the S2 state can quickly equilibrate with another metastable state (S3) where two tyrosine residues, BH residue Y836 and Rpb2 residue Y769, play an important role in stabilizing the DNA TN and RNA 3′-end nucleotide, respectively. Finally, the last transition (from S3 to S4 states) in which the upstream RNA:DNA hybrid backtracks in a single-step, occurring at around hundreds of microseconds, was found to be the rate-limiting step (Fig. 6). Interestingly, one recent study conducted by Imashimizu *et al*.^52^ suggested that the binding of the double-mutant elongation factor TFIIS to the yeast Pol II can result in a paused state of the Pol II complex where rearrangements of the 3′-end RNA nucleotide and the Pol II residues in the active site may take place, and this paused state may finally lead to the backtracking. Related to this experimental finding, we speculate that the frayed state may also play a role in the above-mentioned pausing process. In bacterial RNA polymerase, similar connection has also been suggested by the Landick group^16^.

It has been well documented that the backtracking is sequence-dependent and particularly on the sequence near the RNA 3′-end nucleotide^10^,^53^,^54^. Our mutant MD simulations provided the molecular basis underlying these experimental observations. Our findings indicated that decreasing the strength of the hydrogen bonds within the base pair in the active site could potentially shift the backtracking bias from backtracking-resistant to backtracking-prone by favouring the frayed state. This emphasizes the dominant role of the hydrogen bonds in regulating the backtracking. More intriguingly, the BH bending can continuously examine the base groups of the RNA and DNA nucleotides at the *i*+1 site, which in turn, depending on the stabilities of the base pairs, results in either facilitating the upstream RNA:DNA hybrid to move one register forward (translocation) or the RNA 3′-end nucleotide to fray and consequently lead to backtracking.

Quantitative comparisons of dynamics between our model's predictions and experimental observations are challenging as it is difficult for existing experimental techniques to directly monitor the dynamics of backtracking conformational changes alone. However, recent optical tweezers experiments monitored the dynamics of complete Pol II elongation cycles upon external force, and then they obtained rates of individual conformational changes such as translocation and backtracking by fitting to a kinetic model of the elongation cycle^12^. In this way, they observed that the backtracking rate is significantly slower than the forward translocation (∼13-fold)^12^, which is qualitatively consistent with our simulation predictions (backtracking is ∼5-fold slower than forward translocation^55^). Our model suggests that the slower kinetics of the backtracking is due to the stacking interaction between the RNA 3′-end nucleotide and the Rpb2 residue Y769, which can block the extended backtracking motion. It is also worth noting that their kinetic model was derived based on a completely matched system and the rates they obtained for translocation/backtracking include the TL opening/closing motion, which is not simulated here. Recently, we also fitted a kinetic model with TL opening/closing motion separated from translocation based on both single-molecular and simulation data^56^. Our results suggest that the TL motion is the rate-limiting step of transcription, which explains apparently faster kinetics of simulated translocation and backtracking (∼10–100 μs) compared with single-molecular^12^ and fluorescence studies (∼10 ms)^26^. We also note that the partial DNA scaffold used in our MD simulations may accelerate the dynamics of backtracking. With the recently available structures of full transcription bubble^57^,^58^, we will further investigate this issue in future studies.

In the future, it will be interesting to extend our study to the mismatched pyrimidine base pair, for example, U:T wobble pair, as it may have different dynamic behaviour during the backtracking process compared with the purine base pair because of its smaller molecular size^59^. Second, we can go beyond the one-nucleotide backtracking event and study the backtracking mechanisms for two or even more RNA 3′-end nucleotides, which may give insight into the molecular mechanisms of the transcriptional pausing and arrest^10^,^15^,^55^,^60^. Third, it will also be interesting to investigate the backtracking mechanisms for different mutagenic DNA lesions, such as 8-oxoG and O^6^-methyguanine (O^6^-mdG) and so on, and how some of these lesions can escape Pol II proofreading^61^.

## Methods

We constructed the MSM to investigate the backtracking mechanism in Pol II EC, and our algorithm consists of the following steps: (i) Model the Pol II EC in pre-, frayed and backtracked states; (ii) generate initial low-energy pathways using a modified version of the Climber^49^ algorithm for two proposed backtracking pathways (concerted and stepwise); (ii) seed three rounds of unbiased MD simulations from these initial backtracking pathways and (iv) construct and validate the MSM and further use the MSM to identify metastable intermediate states and obtain both thermodynamics and kinetics.

### System setup and MD simulations

The backtracked and frayed states of Pol II EC were modelled from the X-ray structures of PDB id: 3GTG^8^ and 3HOZ^17^, respectively. The pre-translocation state was built from the above backtracked model by placing an rG:dG mismatched base pair in the active site and removing the backtracked RNA 3′-end nucleotide. The system was first energy-minimized, and then solvated in explicit SPC waters^62^. 77 Na^+^ ions were added to neutralize the system. The final system contains 369,947 atoms. The AMBER force field was used to describe the system (99SB^63^ for protein and metal ions, 99χ (refs 64, 65) for DNA and RNA nucleotides), and all MD simulations were performed at 310K using GROMACS 4.5 (ref. 66; see Supplementary Note 1 for more details of the model construction).

### Generating initial low-energy backtracking pathways

We proposed two models of backtracking: a concerted and a stepwise model. For the concerted model, we applied the Climber^49^ algorithm to generate the pathways along pre-translocation → backtracked states, as well as its reverse route: backtracked → pre-translocation states. For the stepwise model, the initial pathway was produced to follow the route of pre-translocation → frayed → backtracked, and its reverse transition: backtracked → frayed → pre-translocation states. Then, for each backtracking pathway, we geometrically grouped the snapshots from the corresponding Climber simulations into 40 clusters, and 2 random conformations from each cluster were chosen (80 conformations for each model, 160 conformations in total) and were employed for the first round of MD simulations (see Supplementary Note 2 for more details).

### Seeding unbiased MD simulations

In total, we performed four rounds of unbiased MD simulations. In the first round, the above 160 conformations from the initial backtracking pathways were employed as the starting structures for the 10 ns NVT MD simulations. We then selected 80 representative conformations from the first round of MD simulations as the starting structures for the second round of 100 ns MD simulations. Next, we selected another 80 representative conformations from the second round simulations as the starting structures for our third round of 100 ns simulations. To further enhance the sampling, we performed the fourth round of 320 × 100 ns MD simulations by initiating two additional independent simulations with different initial velocities from each of the initial conformations used in our second and third round. Finally, we collected a total of 480 × 100 ns MD simulation trajectories for further analysis (see Supplementary Note 2 for more details).

### Constructing and validating MSMs

To construct the MSM, we followed a two-step procedure^29^: (i) we grouped the MD conformations with similar structures together to generate a set of 800 microstates using the K-centre clustering algorithm^29^; (ii) to visualize the mechanism of backtracking, we further grouped the 800 microstates into 4 macrostates using the Robust Perron Cluster Cluster Analysis (PCCA+) algorithm that is implemented in the MSMbuilder package^31^ (see Supplementary Note 3 for additional details of the MSM construction).

All the reported quantative properties are computed based on the 800-state MSM constructed using the RMSD metrics (Supplementary Figs 8 and 9). To validate our 800-state MSM, we plotted the implied timescale as a function of lag time. The implied timescale plots reach the plateau at the lag time of ∼8 ns (Supplementary Fig. 8b), suggesting our MSM is Markovian at 8 ns or longer lag times. To further validate the model^41^, we predicted the residence probability for a given microstate using our MSM, and these predicted values are in good agreement with those directly obtained from the original MD simulations (Supplementary Fig. 8c).

To ensure that our MSM is independent of the choice of distance metric used for clustering, we built our model based on RMSD as well as the time-structure-based Independent Component Analysis (tICA)^67^,^68^(see Supplementary Figs 10 and 11, 13–15, and Supplementary Note 3 for details). For each method, we grouped the MD conformations (∼4,800,000) into different number of microstates to verify that the constructed MSM is robust to the number of clusters chosen. Notably, both methods render very similar dynamic properties. In particular, the predicted timescale for the slowest transition for the backtracking process was the same for all the MSMs built (Supplementary Figs 9 and 10). Moreover, we observed that the metastable macrostates we identified have consistent structural features regardless of the choice of metrics (see Supplementary Figs 11 and 12 and Supplementary Note 4 for details). We also tested the convergence of our conformational sampling by projecting the sampling from data sets with different sizes (6.4, 19.2, 28.8, 38.4 and 48 μs) onto two slowest tICs. In particular, for each data set, we projected the MD conformations in each microstate separately, and then performed a weighted sum according to their equilibrium populations obtained from the corresponding MSM. As shown in Supplementary Fig. 13, we can clearly see that the projections of the free energy landscape reach reasonable convergence, and particularly no new metastable regions in the phase space was discovered with the increase of conformational sampling. In addition, we also show that MSMs constructed from data sets with different sizes predicted consistent slowest implied timescales (Supplementary Fig. 16). These observations suggest that our MD data set is fairly sufficient to address the backtracking mechanism. We note that the recently developed TRAM method may further improve the quality of MSMs by using thermodynamic data obtained from other enhanced sampling methods to help the construction of MSMs^69^. Moreover, we show that the 4-macrostate MSM is not perfectly Markovian (see Supplementary Figs 17 and 18, and Supplementary Note 3 for details). In this paper, we finally adopted the 800-state MSM, constructed using the RMSD metrics, to report the key metastable states and their associated thermodynamic and kinetic properties.

### MD simulations of matched/mismatched base pair or mutants

We performed additional MD simulations to investigate the backtracking propensity for one matched base pair: rG:dC. In addition, mutant MD simulations were designed to evaluate the functional roles of several critical residues in the backtracking. Finally, to test if the frayed intermediate state is also required for other mismatched base pair systems during the backtracking process, we designed three more Pol II systems with the following base pairs: rA:dG, rC:dT and rU:dT (see Supplementary Note 2 for additional details).

### Generating millisecond trajectories based on our MSM

We used the transition probability matrix at microstate level to generate one 10-ms parallel trajectory, from which we quantitatively calculated several properties, including the RMSD for the backbone movements, cross-correlation values and the RMSF for the BH bending motion. In addition, the average values and the standard deviations of the populations and MFPTs were calculated by bootstrapping the MD trajectories for 100 times. During each bootstrapping, we repeatedly selected a MD trajectory by random from our data set for 480 times with replacement. Then, for each bootstrapped sample, we constructed the MSM and calculated the transition probability matrix at microstate level using a lag time of 8 ns (Markovian time) to generate one 10-ms long trajectory, from which we calculated the stationary distributions of the 4 macrostates and the MFPT for each transition. Finally, the average values and corresponding standard deviations were calculated by averaging the results from all 100 samples.

### Cross-correlation calculations

To study the correlated motions between the DNA TN/RNA 3′-end nucleotide and the Pol II residues, we calculated the cross-correlations for the pairs we are interested in (see Supplementary Note 3 for details).

### TFIIS-dependent transcript cleavage assays

TFIIS cleavage reactions were performed by pre-incubating purified Pol II (WT or Rpb1 T831A mutant) with the RNA:DNA scaffolds as used in the theoretical studies with either rG:dC or rG:dG at *i*+1 site. Reactions were quenched at various time points and the products were separated by denaturing PAGE (see Supplementary Note 5 for more experimental details).

### Intrinsic transcript cleavage assays

Cleavage reactions were performed by pre-incubating purified Pol II (WT or Rpb1 T831A mutant) with the RNA:DNA scaffolds containing either rG:dC or rG:dG bp. The Pol II EC was assembled with the following scaffold in a 20 mM Tris-HCl (pH=7.5) without Mg^2+^.


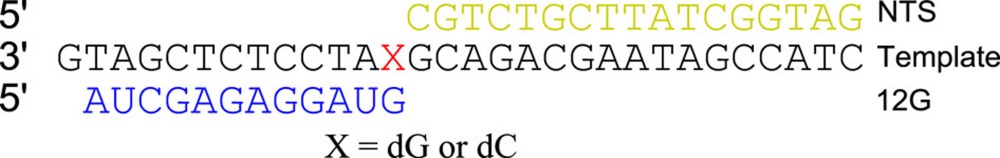


Intrinsic cleavage was initiated after addition of Mg^2+^. Final concentrations for intrinsic cleavage were 20 mM Tris-HCl (pH=9), 100 nM Pol II, 25 nM scaffold and 50 mM MgCl_2_. The reaction was quenched in 0.5 mM EDTA at various time points and analysed by denatured PAGE. Time points in this assay were 1 min, 5 min, 20 min, 1 h, 3 h, 8 h and 24 h. (see Supplementary Note 5 for more experimental details).

## Additional information

**How to cite this article**: Da, L.-T. *et al*. Bridge helix bending promotes RNA polymerase II backtracking through a critical and conserved threonine residue. *Nat. Commun.* 7:11244 doi: 10.1038/ncomms11244 (2016).

## Supplementary Material

Supplementary Information Supplementary Figures 1-18, Supplementary Notes 1-6 and Supplementary References

Supplementary Movie 1 Movie showing dynamics of a complete Pol II backtracking event

## Figures and Tables

**Figure 1:**
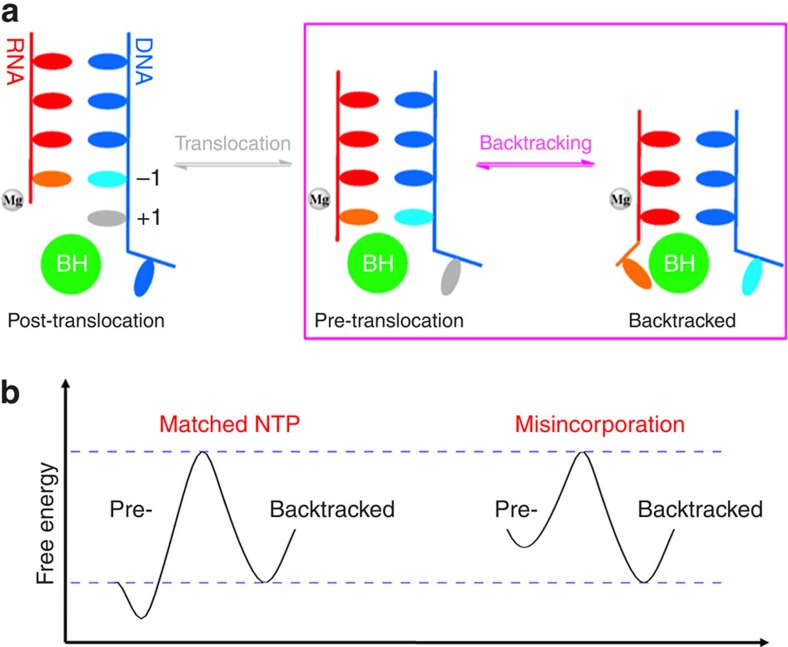
Translocation and backtracking for RNA Pol II Elongation Complex (EC). (**a**) Cartoon models of the translocation and backtracking for RNA Pol II Elongation Complex (EC) during transcription elongation. Upstream DNA (in blue), RNA (in red), Bridge helix (BH, in green), Mg^2+^A (in grey) are shown. In particular, the DNA transition nucleotide (TN) in translocation and backtracking models is highlighted in grey and cyan colour, respectively, and the RNA 3′-end nucleotide in backtracking model is highlighted in orange. (**b**) Schematic free energy landscape for pre-translocation and backtracked states. The transition barrier between these two states will vary under different conditions: matched NTP (left) and mis-incorporation that can be captured by proofreading (right).
