# Supplementary material for: Bridge helix bending promotes RNA polymerase II backtracking through a critical and conserved threonine residue
Source: Nat Commun. 2016 Apr 19;7:11244. doi: 10.1038/ncomms11244 (PMC4838855; doi:10.1038/ncomms11244)
Supplement: Supplementary Information — Supplementary Figures 1-18, Supplementary Notes 1-6 and Supplementary References [file ncomms11244-s1.pdf]

Supplementary Figures

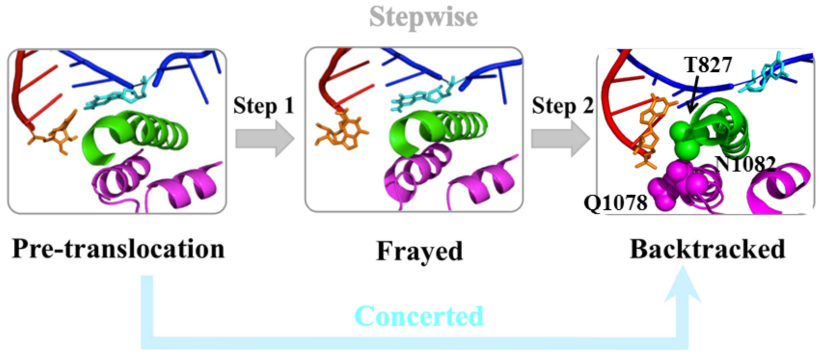

Supplementary figure 1. Two proposed backtracking models in Pol II EC: concerted and stepwise. Three starting structures: pre-, frayed and backtracked states were created based on X-ray structures (PDB id: 3HOZ and 3GTG). The RNA:DNA hybrid (red/blue), bridge helix (green) and trigger loop (purple) are shown. The RNA 3'-end nucleotide and the DNA transition nucleotide are highlighted with stick models colored in orange and cyan respectively.

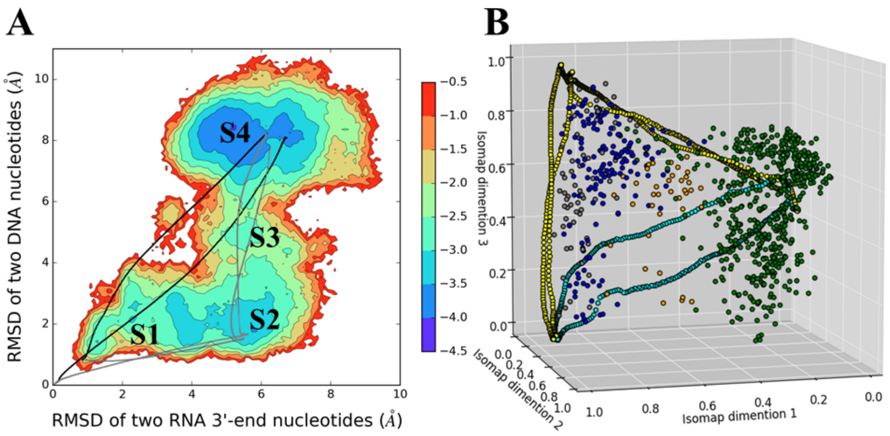

Supplementary figure 2. **Initial pathways obtained by the Climber algorithm do not determine the MSM result.** (a). The conformations derived from the biased simulations were projected onto two reaction coordinates: RMSD of the last two RNA 3'-end nucleotides and RMSD of their base-paired DNA nucleotides, using the pre-translocation state as the reference (unit in kcal/mol). The locations of the four metastable states (S1-S4) are also labeled. The conformations along the initial pathways produced by the Climber algorithms are also superimposed. Initial pathways (in both forward and backward directions) belonging to the concerted and stepwise mechanism are shown in black and gray lines respectively. (b). Projection of the MSM microstates and Climber trajectories onto the three dominant components identified by the Isomap dimensionality reduction technique. For each microstate, 10 random conformations were chosen, and the results were illustrated by 4 macrostates: S1 (gray), S2 (blue), S3 (orange) and S4 (green). The conformations from the initial pathways were also mapped onto the same components: concerted and stepwise pathways are shown in cyan and yellow, respectively.

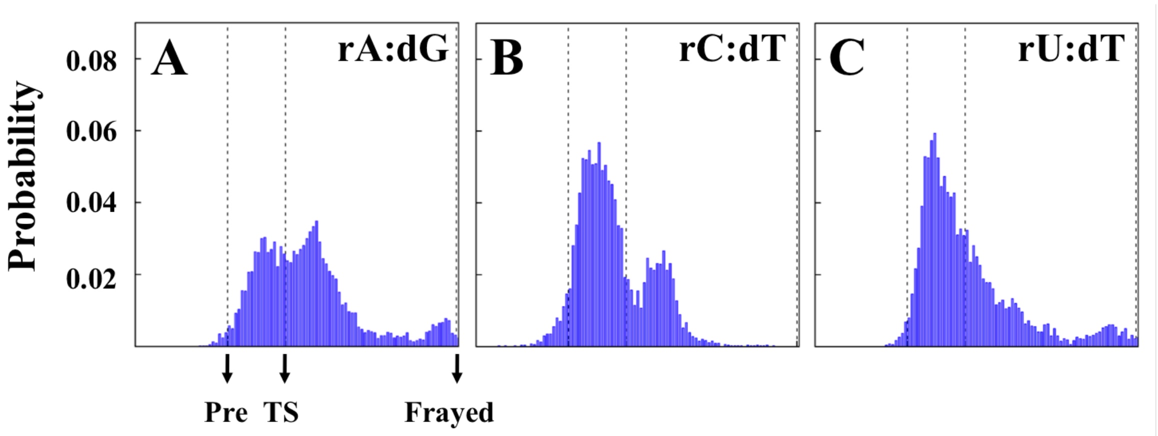

Supplementary figure 3. Frayed state is also a likely intermediate state during the backtracking process for other mis-matched base pairs, including rA:dG (a), rC:dT (b) and rU:dT (c) base pairs. The starting structures were selected from the region near to the Transition State (TS) between the pre- and frayed states in the rG:rG mismatched system (corresponding to the region around the RMSD coordinate (3, 2.5) as shown in the free energy profile in Supplementary Fig. 2a).

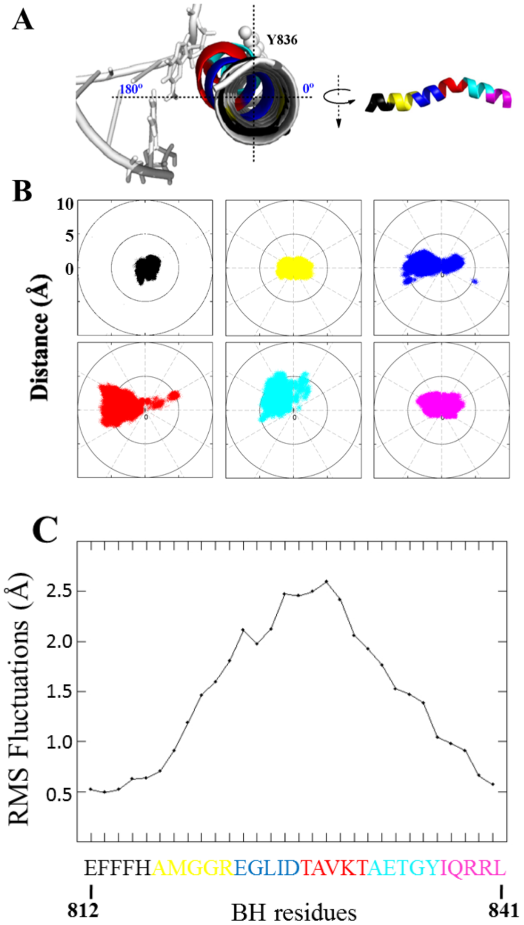

Supplementary figure 4. **The directionality and intensity of the Bridge Helix (BH) bending motion**. To describe the bending motion of the BH, we firstly built a perfect helix structure and superimposed it to the pre-translocation state by fitting the C_α atoms of three helical turns (black, yellow and purple turns in (a)). We defined a polar axis using the center of the perfect helix and its residue Y836. (a). In this polar coordinate, the origin is the center of the perfect helix, and the line connecting the origin and the center of mass of Y836 (from perfect helix) is seen as the vertical axis. (b). We mapped all the conformations from the MD simulations onto this polar axis for each of the six helical turns of the BH (from residues E812 to L841, colored in black, yellow, blue, red, cyan and purple respectively). We also calculated the distance between the center of mass of each turn and the origin point. (c). The RMS Fluctuation values of the C_α atoms of the BH residues averaged over the conformations from one 10-millisecond trajectory. The residues belong to the helical turns with the same color shown in a.

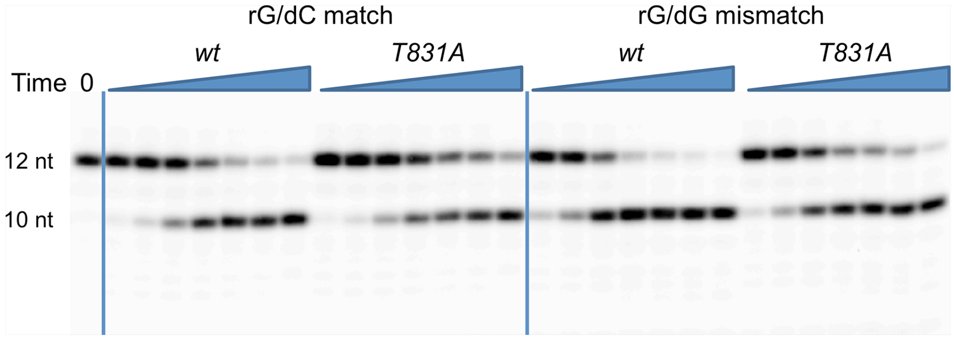

Supplementary figure 5. **Intrinsic cleavage assay of RNA Polymerase II**. Scaffolds and the conditions used here are the same as those used in Fig. 3, except that pH was adjusted to 9, and the concentration of Mg^{2+} was increased to 50 mM. Time points are 1 min, 5 min, 20 min, 1 hr, 3 hr, 8 hr and 24 hr. The upper bands refer to the initial RNA transcript (12nt); the lower bands refer to the cleaved product (10nt).

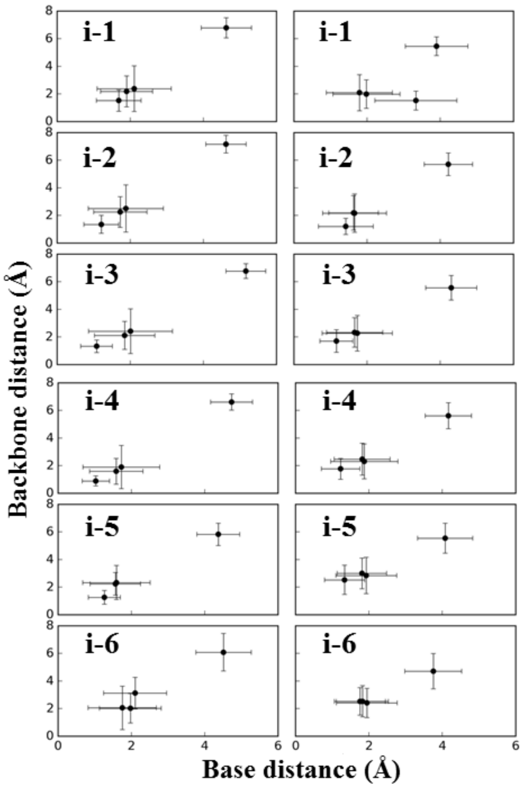

Supplementary figure 6. The distance to the pre-translocation state for the backbone phosphate and the nucleotide base of the individual upstream DNA (left panel) and RNA (right panel) nucleotides (From i-1 to i-6 sites). The fluctuations of the distances within each state are indicated with error bars.

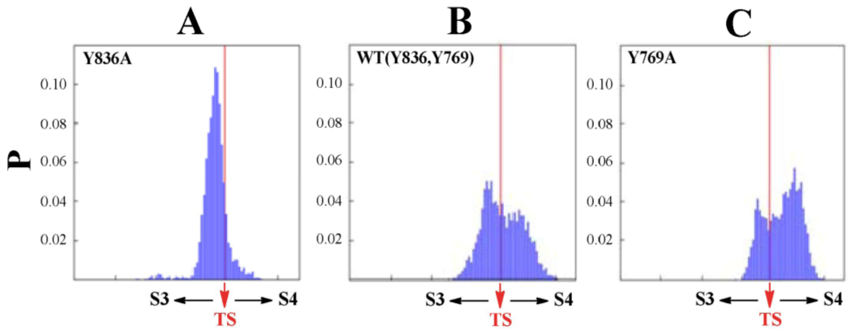

Supplementary figure 7. Evaluations of the functional roles of the BH residue Y836 and Rpb2 residue Y769 in backtracking dynamics. Y836 can facilitate the transition of the DNA TN from S3 to S4 states through direct packing interaction between the base group of TN and aromatic ring of Y836. Starting from the region close to the TS between S3 and S4 state, the Y836A mutation prevents the transition of the DNA TN to the S4 state (a) compared with the WT (b). However, Rpb2 residue Y769 can block the S3 to S4 transition by stabilizing the frayed RNA 3′-end nucleotide in S3 state, substituting the Y769 with alanine promotes the backtracking (c). The transition index between the S3 and S4 states is defined as X axis.

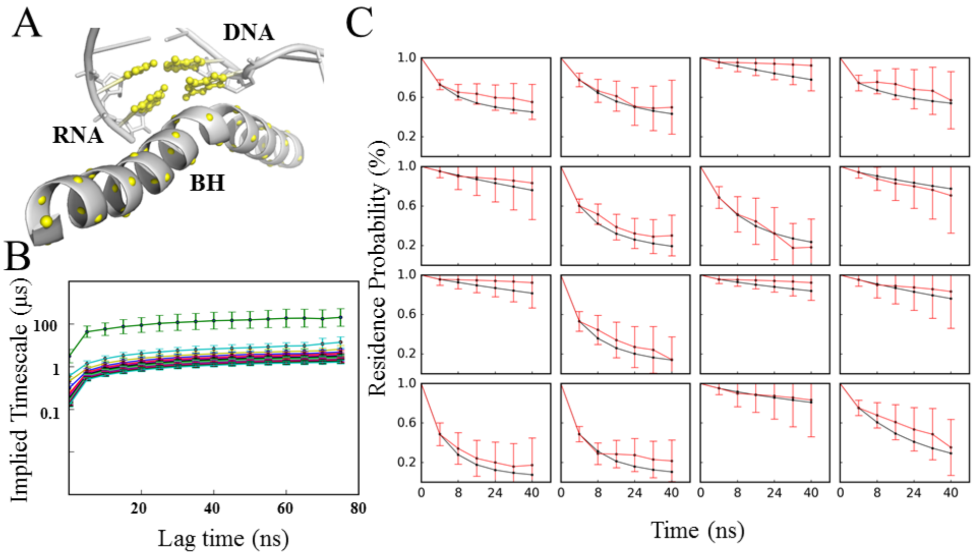

Supplementary figure 8. Construction and validation of the MSM. (a). The atoms used in the RMSD calculations are highlighted in yellow spheres, which include 36 C_α atoms from the Bridge Helix domain (residues Gln811 to Glu846), and heavy base atoms of the two 3'-end RNA nucleotides and their base-paired DNA nucleotides. (b). The implied timescale plot against different lag times for the 800-microstates MSM. The curves level off at ~8ns lag time. The errors of the implied timescales were estimated by generating 100 bootstrapped samples, each by randomly selecting 480 trajectories from the MD simulation trajectories. Then for each bootstrap sample, we constructed the MSM and calculated the corresponding implied timescales. Finally, the average value and the errors were estimated. (c). Validation of the MSM by comparing the probability for the system to remain in a certain microstate as a function of time obtained from the propagation of the MSM (black) and the original MD trajectories (red) using 16 microstates.

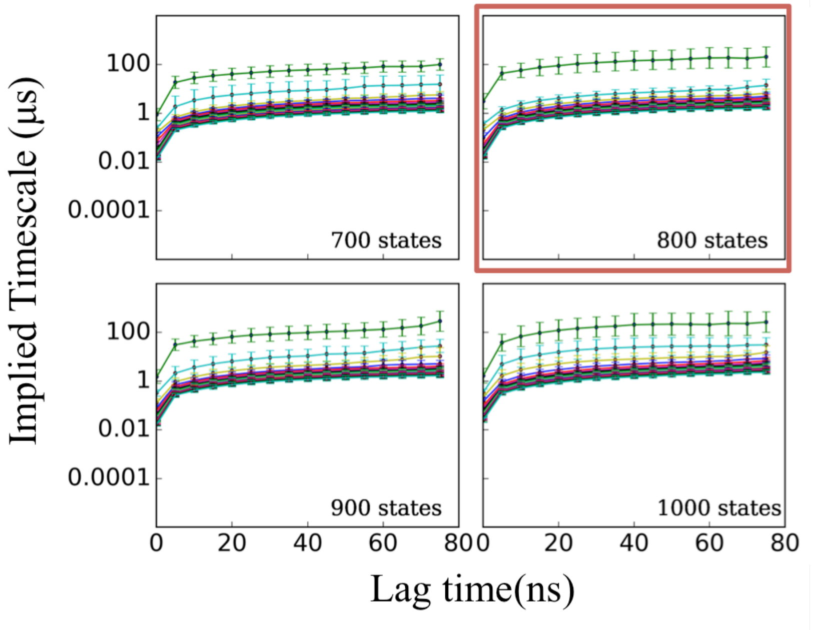

Supplementary figure 9. **Construction of MSMs based on the complete MD simulations dataset with an aggregated time of ~50 microseconds using the RMSD metric.** The implied timescale plots as a function of lag time for MSMs containing different number of states: 700, 800, 900, and 1000, all these MSMs are constructed based on the new dataset (480x100ns MD trajectories). For all the models, the implied timescale curves level off at ~8 ns lag time, and the slowest timescale reaches ~100 μs. The final model used for all the analyses is highlighted by a red frame (800-states MSM with a lag time of 8 ns).

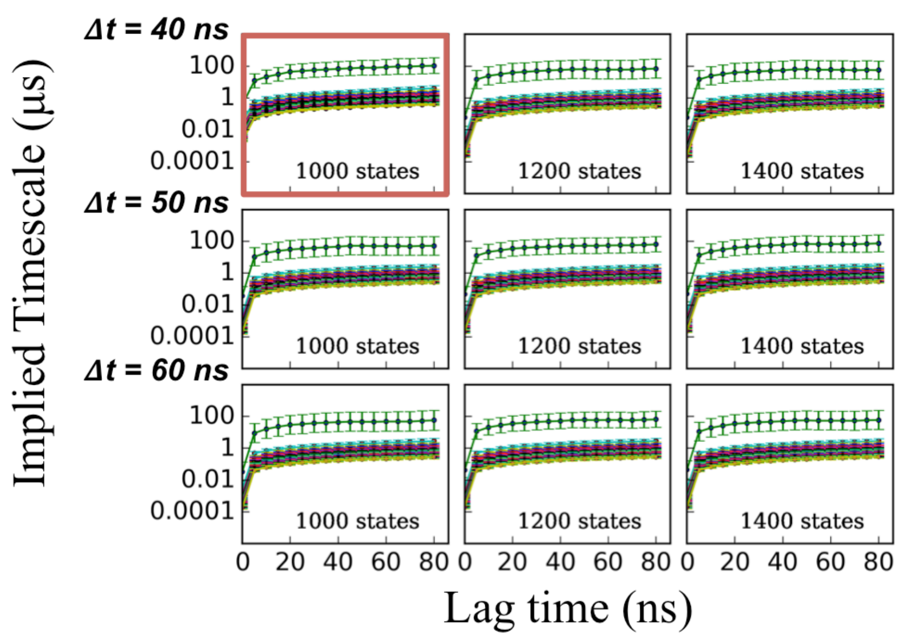

Supplementary figure 10. The implied timescale plots as a function of lag time for the MSMs constructed by projections of MD conformations onto four slowest tICs followed by the K-Centers clustering. From the top to the bottom row, results from tICA analysis with different correlation lag times (Δt): 40ns, 50ns and 60ns are compared. At each correlation lag time, implied timescales predicted by MSMs with different number of states (1000, 1200 and 1400, respectively) are displayed.

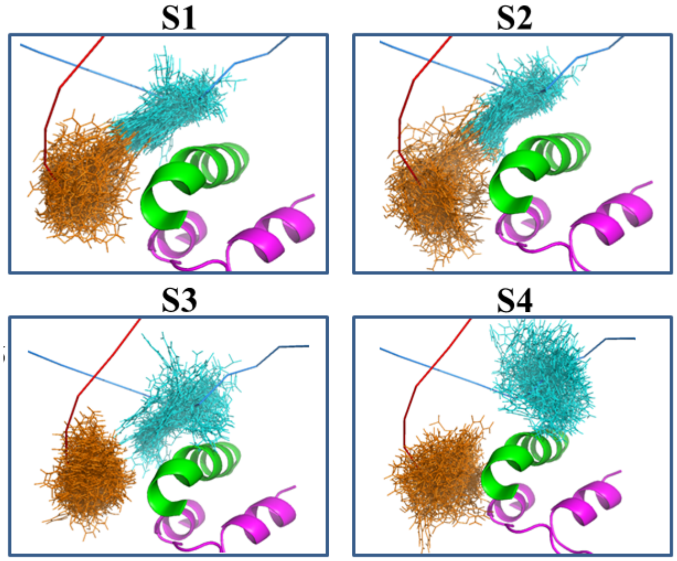

Supplementary figure 11. 100 randomly chosen conformations from each macrostate from a macrostate MSM lumped from a 1000-state microstate MSM constructed based on the tICA analysis. The 3'-end RNA nucleotide and its paired DNA transition nucleotide are represented with orange and cyan sticks, respectively. Please refer to model highlighted by the red frame in Supplementary Fig. 10 for additional details.

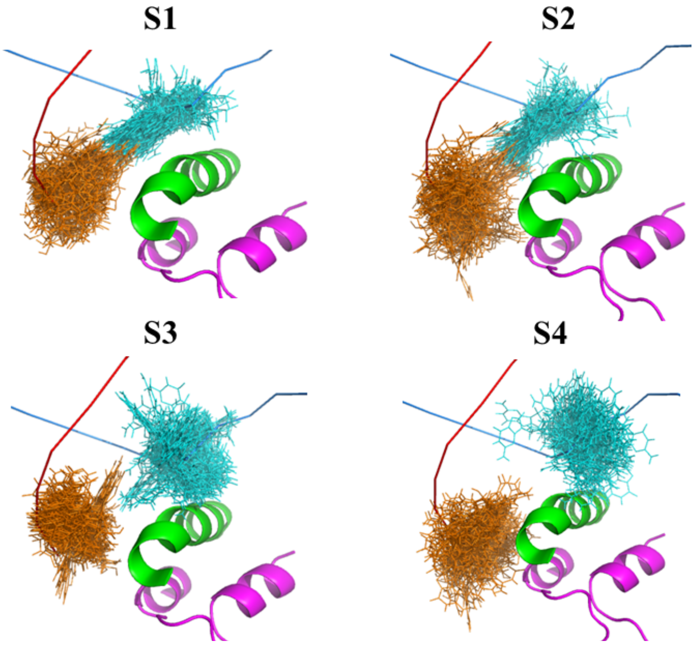

Supplementary figure 12. 100 randomly chosen conformations superimposed for each macrostate (S1-S4) for the MSM built based on the RMSD metric. The 3'-end RNA nucleotide and its paired DNA transition nucleotide are represented with orange and cyan sticks respectively. Bridge helix (green) and trigger loop (magenta) are shown in cartoon representation.

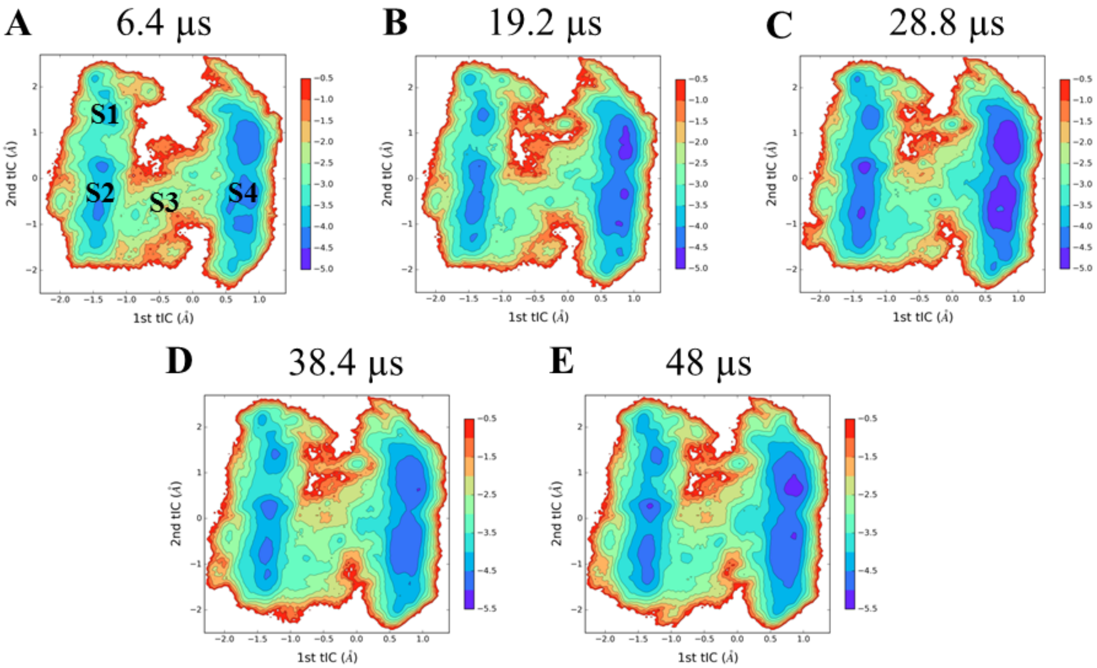

Supplementary figure 13. Convergence of the free energy landscapes was validated by projecting the subsets of the MD conformations (aggregated time of 6.4µs, 19.2µs, 28.8µs, 38.4µs and 48µs) onto two slowest tICs (unit in kcal/mol).

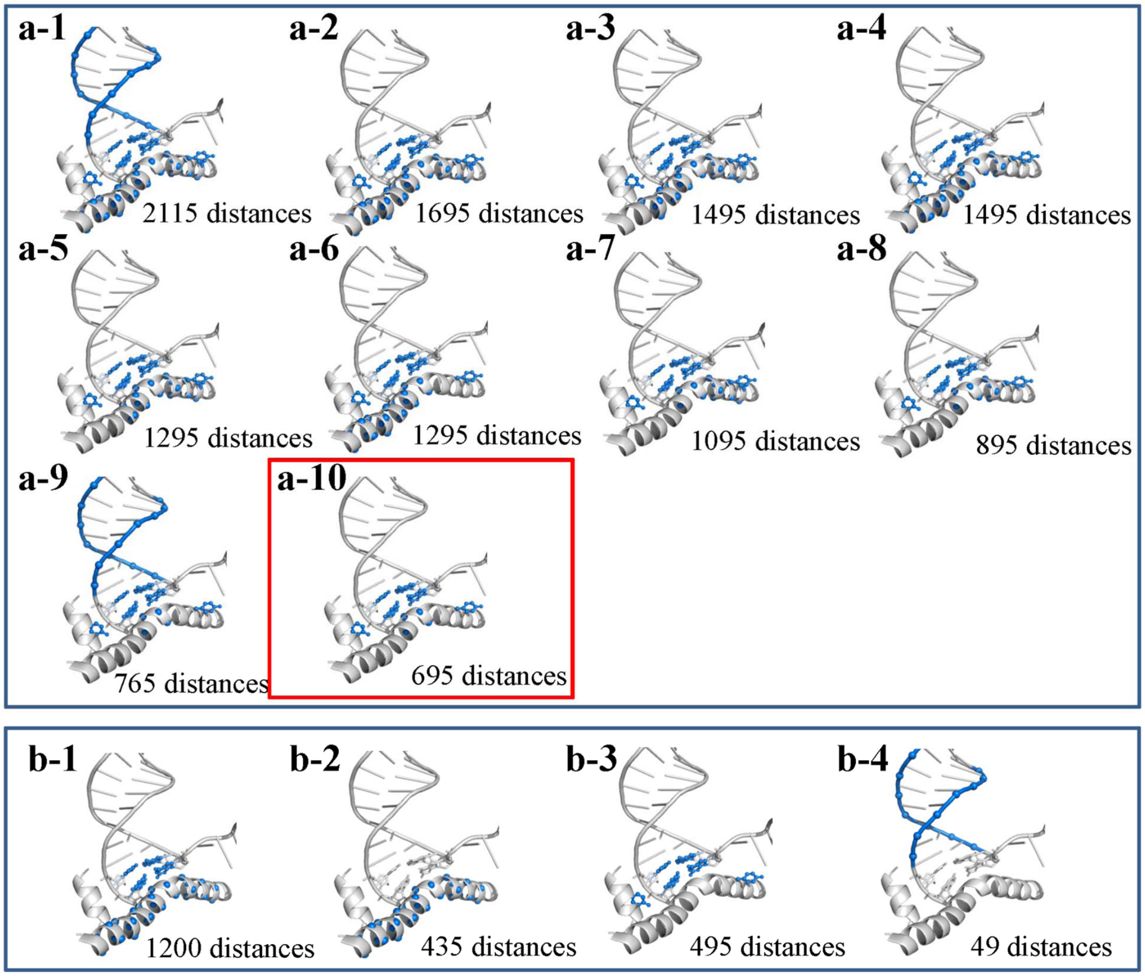

Supplementary figure 14. (**a1-a10**). Different sets of distance metrics (each consisting of distances between a group of atom pairs, highlighted as blue spheres) to be included in the tICA analysis. The set (**a-10**) was used in our final model, highlighted by the red frame. Sets **(a-1) to (a-9)** and **(b-1) to (b-4)** contain difference set of distances in order to examine if distances included in (a-10) are sufficient and necessary to describe the conformational dynamics of Pol II backtracking. Please refer to the text for the details of each set of atom pairs.

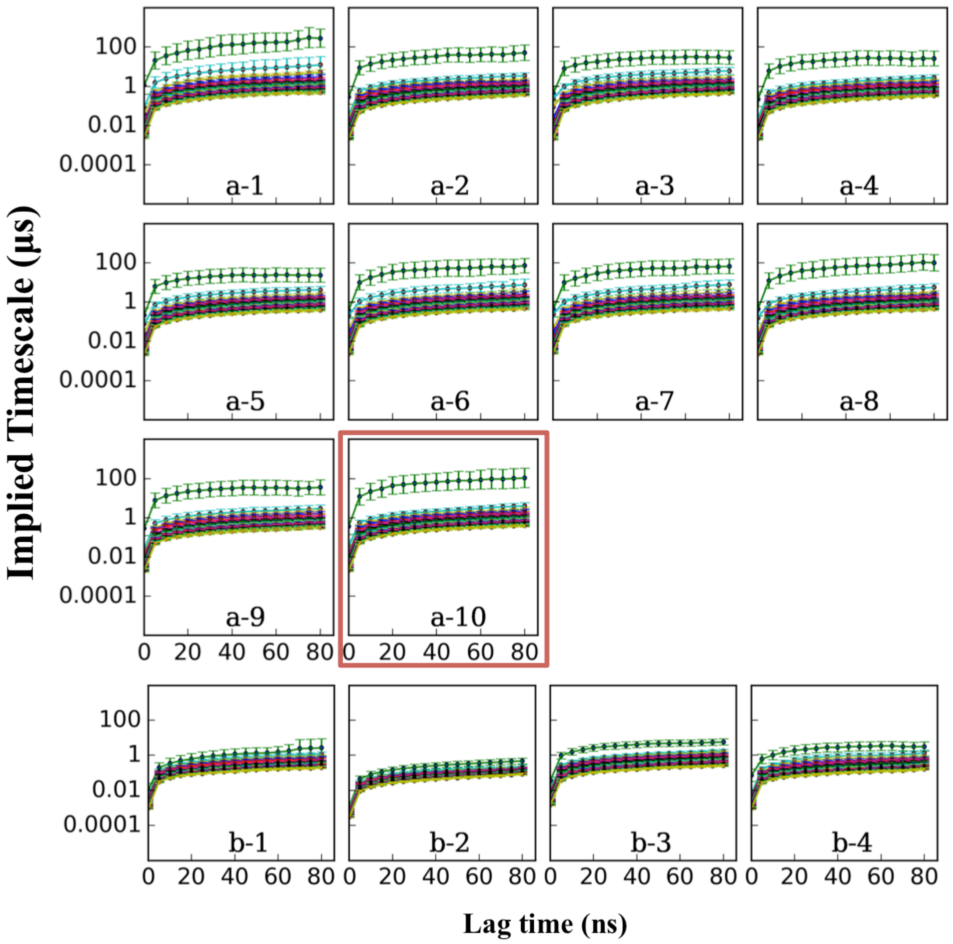

Supplementary figure 15. The implied timescale plots as a function of lag time for the MSMs constructed using slowest tICs identified by the tICA analysis based on the corresponding distance sets shown in Supplementary Fig. 14. For each set, an MSM was constructed by projections of MD conformations onto 4 slowest tICs followed by the K-centers clustering to produce 1000 states. The correlation lag time for the tICA analysis was chosen to be 40 ns, and the lag time for MSMs are set as 8 ns.

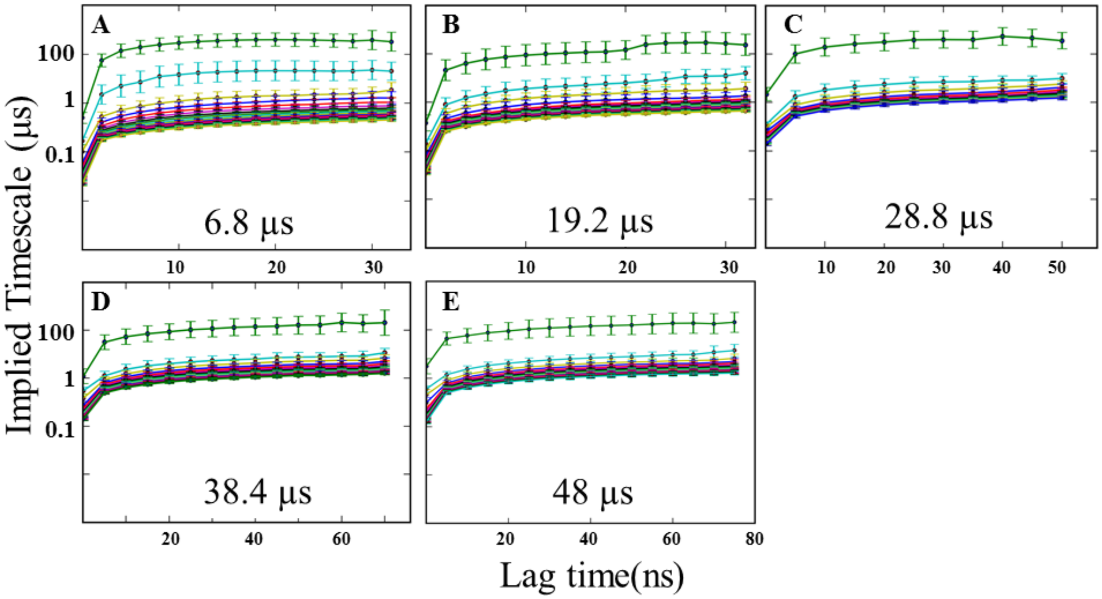

Supplementary figure 16. Implied timescales as a function of lag time predicted by MSMs constructed based on the RMSD metric from five MD datasets with an aggregated simulation time of: (a) 6.8 μs (160 trajectories × 40 ns), (b) 19.2 μs (480 trajectories × 40 ns), (c) 28.8 μs (480 trajectories × 60 ns), (d) 38.4 μs (480 trajectories × 80 ns), (e) 48 μs (480 trajectories × 100 ns). These MSMs contain 120, 500, 500, 800, and 800 states, respectively. The errors of the implied timescales were computed by generating 100 bootstrapped samples, each by randomly selecting 480 trajectories with replacement from 480 MD trajectories.

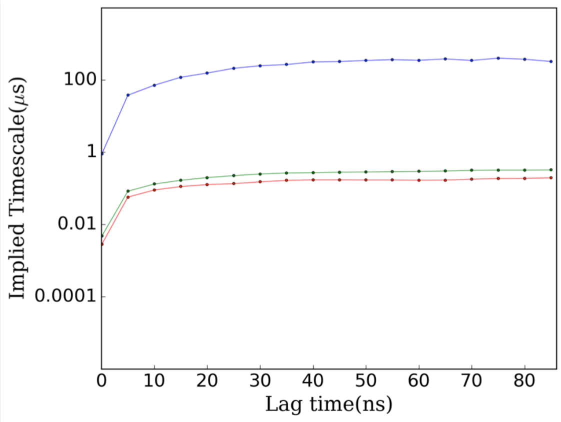

Supplementary figure 17. Validation of the MSM at macrostate level: plot of implied timescale against lag time for the 4-macrostate MSM. The curves level off at ~30ns lag time.

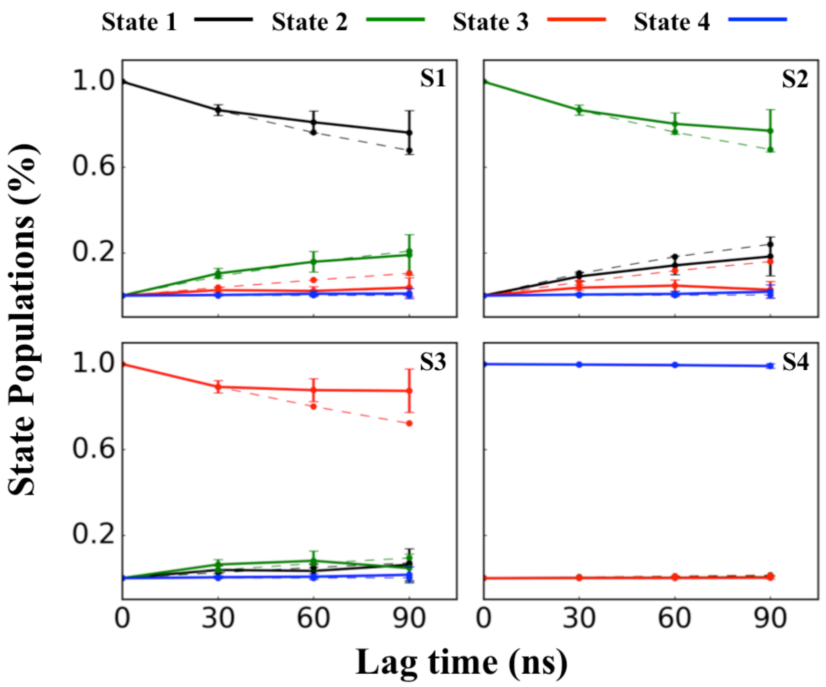

Supplementary figure 18. Examining the Markovianity of the macrostate MSM by the Chapman–Kolmogorov test. Population of the macrostates as a function of time obtained from the propagation of the MSM (dashed line) and the original MD trajectories (solid line). The MSM was constructed at a lag time of 30 ns.

Supplementary Notes

Supplementary note 1. System Setup.

Modeling the backtracked Pol II EC. The backtracked Pol II Elongation Complex (EC) was modeled from the recent X-ray structure (PDB id: 3GTG)[1] where the RNA 3′-end nucleotide is in backtracked form. The missing loops of Pol II were rebuilt using the free software SWISSpdb[2]. The remaining parts of the system, including the RNA:DNA hybrid, downstream DNA duplex, 8 Zn^{2+} ions and one Mg^{2+} ion were all kept. Finally, in order to make the system size the same as the pre-translocation (pre-) and frayed states, we removed the last base pair (bp) from the RNA:DNA hybrid.

Modeling the pre-translocation and frayed states of Pol II EC. To build the pre-translocation state of Pol II EC, we shifted the DNA and RNA chains in the above-modeled backtracked Pol II EC by one bp towards the upstream direction and mutated the nucleotides accordingly, and then we removed the backtracked RNA 3′-end nucleotide. We modeled the active site RNA nucleotide in a syn form and its pairing DNA nucleotide in an anti form so that this bp can form the syn-anti wobbled base pair. This is because: (1) In the DNA polymerase systems, it has been observed that the dG (from product chain)-dG (from template chain) mismatched base pair at the active site adopts a syn-anti wobbled state[3]. (2) It is more difficult for the template DNA nucleotide to transit from anti- to the syn-form due to the steric clashes with the nearby protein motifs (such as the BH domain in Pol II) compared to the substrate NTP that has more space for the transition. Finally, we re-modeled the TL domain to the fully open state based on the crystal structure (PDB id: 2YU9)[4]. Using this pre-translocation state structural model, we built the frayed Pol II EC by directly replacing the RNA 3′-end Guanine in the active site

with the frayed RNA nucleotide (rG) with its conformation taken from the crystal structure (PDB id: 3HOZ)[5].

Exploring the initial low-energy backtracking pathways. In order to obtain the initial backtracking pathways, we employed the modified version of Climber algorithm[6] to explore the low-energy conformations between the pre- and backtracked states of Pol II EC. In this algorithm, an external restrain potential is applied to a set of coordinates in the initial state and gradually pulls the system to the new set of coordinates in the final state. The strength of the applied potential is self-adjusted so that the system can transit along a low-energy pathway. Here we proposed two backtracking models: the concerted and the stepwise model. For the concerted model, we performed the Climber simulations directly from the pre- to backtracked states as well as the reverse transition. For the stepwise model, the Climber simulations were firstly conducted from the pre- to frayed states, and then from frayed to backtracked states, and also the reverse transitions. The restrain potentials were applied to the nucleic acids only. We set the target number of morphing cycles to 750, where each morphing cycle consisted of 100 iterations of morphing with 20 steps of conjugate gradient-energy minimization each 10 morphing iterations. Finally, for each backtracking pathway, we geometrically grouped the snapshots from the corresponding Climber simulations into 40 clusters using the K-center clustering algorithm implemented in MSMbuilder software[7]. Finally we selected 2 random conformations from each cluster for the following unbiased MD simulations (160 conformations in total, 80 conformations for each model).

Supplementary note 2. Setup of the MD simulations.

All the MD simulations were performed using GROMACS 4.5 package[8]. The Pol II EC was centered in a dodecahedron box, and the minimum distance between the system and the box wall was set to 7.0Å. The system was then solvated in SPC water[9] and 77 Na^+ ions

were added to make the whole system neutral. The final system contains 369,947 atoms. The AMBER force field was used to describe the system (for protein and metal ions 99SB[10]and for DNA and RNA nucleotides 99χ[11,12] that was designed to correct the parameters of the glycosidic dihedral angle). A switched Van der Waals type was used with a cutoff of 11Å. Short-range electrostatic interactions were cut off at 12Å and long-range electrostatic interactions were treated with the Particle-Mesh Ewald (PME) summation method[13,14]. All the chemical bonds were constrained using the LINCS algorithm[15]. The energy minimization was conducted using the steepest decent minimization method, followed by 120-ps restrained MD simulations by constraining the heavy atoms of the proteins, DNA and RNA chains. Then, temperature annealing was performed (only for the first round of MD simulations) by increasing the system temperature from 50K to 310K within 200ps. Finally, with a time step of 2fs, the NVT production MD simulations were collected at 310K using the velocity rescaling thermostat[16].

In total, we performed four rounds of MD simulations. In the first round (non-production MD simulations), 80 snapshots from the morphing simulations for each proposed backtracking pathway were chosen and used as the starting structures for the following 10ns NVT MD simulations, finally a total of 160 10-ns MD trajectories were collected. Next, with the conformations from the last 5ns of each MD simulation in the first round, we geometrically grouped them into 80 clusters using the K-centers algorithm. The RMSD of the non-hydrogen atoms of the DNA TN and RNA 3'-end nucleotide was used as the distance function for the clustering, and before each calculation, the conformation was firstly fitted to the minimized pre-translocation state by the C_α of the proteins. Then one conformation was randomly selected from each cluster and used as the starting structures to seed the 2nd round of 80 100-ns MD simulations. Similarly, we have further seeded the 3rd round of 80 100-ns MD simulations. To further enhance the sampling, we performed the 4th round of 320 100-ns MD simulations by initiating two additional independent simulations

with different initial velocities from each of the initial conformation of our 2nd and 3rd round. Finally, we collected a total of 480 100-ns production MD simulations that were used to build the final MSM and for data analysis.

MD simulations of Pol II EC with different base pairs or specific residue mutants. In order to study the backtracking propensity for different base pairs, we firstly designed one additional completely matched base pair (rG:dC) for MD simulations. In addition, we also designed three other mismatched bp systems: rA:dG, rC:dT and rU:dT. Seven representative conformations for the MD simulations were selected from the region near to the Transition State (TS) between the S1 and S2 states of the rG:dG system (corresponding to the region around the RMSD coordinate (3, 2.5) in the free energy profile in Supplementary Fig. 2a). Then for each conformation, we substituted the RNA 3′-end nucleotide (rG) and the DNA TN (dG) bp to the rG:dC, rA:dG, rC:dT and rU:dT bp respectively. We used the seven TS conformations selected above as starting structures, but replacing the rG and dG to the corresponding RNA and DNA nucleotides. Then, we performed 10-ns MD simulations for each of the above newly generated conformations with five different base pairs (rG:dC, rG:dG, rA:dG, rC:dT and rU:dT) using the same setup for the MD simulations described in previous section. Furthermore, to evaluate the functional roles of the BH residue T831 on the rG:dC and rG:dG systems, the same TS conformations chosen above were used for the T831A mutant MD simulations (seven 10-ns MD trajectories from each bp system were collected for data analysis).

Finally, we further evaluated the functional roles of other two Pol II residues: Y836 and Y769 in the backtracking transition from S3 to S4 states. Four starting points for the subsequent WT and mutant (Y836A and Y769A) 10-ns MD simulations were chosen from the TS region between S3 and S4 states (with the RMSD value of ~6.3Å along the Y-axis in the free energy profile in Supplementary Fig. 2a). To generate the mutants, we used

PyMOL[17] to mutate the residue Y836 to the desired amino acids followed by a visual inspection to select the best rotamer. A total of 12 10-ns MD trajectories were collected for data analysis.

Supplementary note 3. Construction of the Markov State Model (MSM)

MSMs partition the conformational space into a set of metastable macrostates[7,18-21], each corresponding to an energy minimum in the free energy landscape, so that the transitions within each macrostate are fast but the inter-macrostate transitions are relatively slow. With a given lag time Δt that is longer than the intra-state relaxation time, the fast transitions within each macrostate can be integrated together, which ensures the MSM is markovian. That is, the distributions of the state $j(t+\Delta t)$ after each lag time Δt only depend on the distributions of the states at the time t, $j(t)$, but not on any other states. In this probabilistic manner, the long time dynamics can be easily propagated using the following equation:

$$P(n\Delta t) = [T(\Delta t)]^n P(0) \quad (1)$$

Where $P(n\Delta t)$ is a vector of state distributions at time $n\Delta t$ and T is the transition probability matrix with the lag time of Δt.

In this work, we followed a splitting and lumping procedure to build our MSM:

Splitting the conformations into microstates.

We performed geometric clustering for the MD conformations (~4800K) into various number of cluster size using two different metrics: RMSD and the time-structure based Independent Component Analysis (tICA).

<u>*Clustering using RMSD*</u>. We grouped all the MD conformations (~4800K) into 700, 800, 900, 1000 clusters respectively using the K-centers algorithm. The distance was defined as the RMSD of 36 C_α atoms from the BH (residues Gln811 to Glu846), and all the heavy

base atoms of the two RNA 3'-end nucleotides and their corresponding paired DNA nucleotides (highlighted with yellow spheres in Supplementary Fig. 8a). For each cluster size, we then calculated the transition probability matrix $T(\Delta t)$. The transition count N_{ij} from state i to j was firstly obtained by counting the number of transition between these two pairs in the MD simulations at a certain lag time Δt, then symmetrizing the count matrix and normalizing each row by:

$$T_{\mathrm{ij}} = N_{\mathrm{ij}} / \sum_{\mathrm{j}} N_{\mathrm{ij}}$$

Calculating the implied timescales and determining the Markovian lag time. We plotted the implied timescale as the function of the lag time, calculated using the following equation:

$$\tau_{\mathrm{k}} = -\frac{\tau}{\ln \mu_{\mathrm{k}}(\tau)}$$

where μ_k is the eigenvalue of the transition probability matrix T with the lag time τ. Each implied timescale indicates the average transition time between two groups of states, that is, the slow τ_k describes the slowest dynamics, which likely corresponds to the principal conformational change of interest. For the MSMs constructed using the RMSD metric, all the implied timescale plots consistently predict the same slowest timescales regardless of number of states the MSMs contain (see Supplementary Fig. 9).

tICA analysis. In order to check if the model and the backtracking mechanism are independent of the choice of metric, we rebuilt our model using time-structure based Independent Component Analysis (tICA). In particular, we select distances between the following atom pairs to include in the tICA analysis (see Supplementary Fig. 14 (a-10)):

i±1 site DNA & RNA nucleotides (heavy atoms on the base) - i±1 DNA & RNA nucleotides (heavy

atoms on the base) (except i-1 DNA - i-1 RNA atom pairs)
i±1 site DNA & RNA nucleotides (heavy atoms on the base) – 5 BH residues (Thr827 to Thr831, Cα atoms)
i+1 site RNA nucleotide (heavy atoms on the base) - Rpb2 residue Y769 (side-chain heavy atoms)
i+1 site DNA nucleotide (heavy atoms on the base) – Rpb1 residue Y836 (side-chain heavy atoms)

The above set of atom pairs contain backtracked RNA and DNA nucleotides, critical Bridge Helix (BH) residues, and two Tyr residues (Y769 and 836) which are shown to stabilize the nucleotide bases during backtracking. In our mechanism, distances between these atom pairs are sensitive to the backtracking of Pol II. Based on these distance metrics, we projected MD conformations onto 4 slowest tICA components, and then applied the K-centers algorithm[7] to cluster conformations into different number of states (1000, 1200 and 1400 states respectively) and also under different correlation lag times (40ns, 50ns and 60ns, respectively). Notably, selection of the top 6 tICA components will not change the model (data not shown). Finally, for each model above, we then constructed the MSM and plotted the corresponding implied timescales (see Supplementary Fig. 10 and discussions below).

We further show that the above set of atom pairs we selected based on physical intuitions is also consistent with the optimal set picked by tICA in an unbiased way. In particular, we applied tICA to choose distance metrics that can best describe the slowest dynamics corresponding to the backtracking of Pol II. To achieve this, we include different sets of distances (by varying atom pairs from protein residues and DNA/RNA nucleotides) in the tICA calculations, and further examine whether MSMs constructed based on slowest tICs can correctly predict implied timescales of backtracking.

We chose 14 different sets of distance metrics, each consisting of distances between a number of atom pairs. In particular, nine sets of atom pairs shown in Supplementary Fig. 14

(a-1) to (a-9) were designed to examine if atom pairs included in our model (Supplementary Fig. 14 (a-10)) are sufficient to describe the slowest dynamics of backtracking. To achieve this, we have systematically introduced additional atom pairs to create new sets as shown in Supplementary Fig. 14 (a-1) to (a-9).

- For atom sets (a-9) and (a-1), we include additional atom pairs from other RNA and DNA nucleotides to examine if these additional nucleotides may significantly impact the backtracking dynamics:

(a-9): i-2 site to i-8 site DNA & RNA nucleotides (P atoms) - 5 BH residues (827 to 831, Cα atoms)
(a-1): i-2 site to i-8 site DNA & RNA nucleotides (P atoms) - 30 BH residues (812 to 841, Cα atoms)

- For atom sets (a-2) to (a-8), we include additional atom pairs from more BH residues to examine if other parts of this critical Pol II motif may contribute substantially to the backtracking dynamics:

(a-8): i±1 site DNA & RNA nucleotides (heavy atoms) - 10 BH residues (827 to 836, Cα atoms)
(a-7): i±1 site DNA & RNA nucleotides (heavy atoms) - 15 BH residues (822 to 836, Cα atoms)
(a-6): i±1 site DNA & RNA nucleotides (heavy atoms) - 20 BH residues (812 to 831, Cα atoms)
(a-5): i±1 site DNA & RNA nucleotides (heavy atoms) - 20 BH residues (822 to 841, Cα atoms)
(a-4): i±1 site DNA & RNA nucleotides (heavy atoms) - 20 BH residues (822 to 841, Cα atoms)
(a-3): i±1 site DNA & RNA nucleotidse (heavy atoms) - 25 BH residues (822 to 841, Cα atoms)
(a-2): i±1 site DNA & RNA nucleotides (heavy atoms) - 30 BH residues (812 to 841, Cα atoms)

For each set, an MSM was constructed (via projections of MD conformations onto 4 slowest tICs followed by K-centers clustering into 1000 states) and the implied timescales were calculated. As shown in Supplementary Fig. 15 (a-1) to (a-10), the introductions of additional atom pairs from various parts of system in tICA analysis do not have substantial impact on the predicted slowest implied timescales. These results suggest that atom pairs included in our model are sufficient to describe the conformational dynamics of the backtracking process.

Furthermore, we also designed additional sets to examine if all atom pairs included in our model are necessary to describe the slowest dynamics of backtracking. To achieve this, we have removed fractions of atom pairs from set (a-10) to compile 4 additional sets as shown in Supplementary Fig. 14b:

- (b-1): We only include atom pairs between nucleotides and BH.
- (b-2): We only include atom pairs from BH.
- (b-3): We only include atom pairs from i+1 site DNA and RNA nucleotide, and two Tyr residues (Y769 and 836).
- (b-4): We only include atom pairs from upstream RNA and DNA nucleotides.

As shown in Supplementary Fig. 15b**,** exclusion of any of the three critical components, i+1 RNA&DNA nucleotides, BH, and two Tyr residues, will significantly accelerate conformational dynamics, with the predicted slowest implied timescale decreased from ~100 μs to ~1 μs. These results indicate that atom pairs included in our model are essential to ensure that our MSMs can properly model the conformational dynamics of backtracking.

Convergence of MSMs constructed from different subsets of MD simulations. We further examine if the implied timescales predicted by MSMs constructed from different MD simulation datasets (containing an aggregated simulation time of 6.4 μs, 19.2 μs, 28.8 μs, 38.4 μs, and 48 μs) are converged. To better compare different sets of implied timescales, we have also reported their uncertainties obtained from the bootstrapping method. As shown in Supplementary Fig. 16, the implied timescales from different MSMs are all within one standard deviation, and level off at ~100 μs. These results suggest that our MSMs reached a reasonable convergence in predicting the slowest dynamics in Pol II backtracking.

Validating the 800-state MSM. We have validated the MSM using residence probability functions[20,22]. In particular, we show that the time evolution of state populations predicted by propagation of the MSM matches well with those directly obtained from MD trajectories (see Supplementary Fig. 8c).

Taken together, the above results indicate that our model is robust to choice of clustering metric and number of clusters. Therefore, we decided to use the 800-state model obtained from the RMSD metrics to obtain the metastable states and their associated thermodynamic and kinetic properties. In this model, the implied timescale plot levels off after ~8ns lag time (see Supplementary Fig. 8b), suggesting that the model is markovian at this or longer lag times. Hence we set the lag time as 8ns to build our MSM and compute all the quantitative properties.

Lumping microstates into four macrostates. To visualize the metastable states, we further grouped the 800 microstates derived from the clustering based on the RMSD metric into 3 macrostates using the Robust Perron Cluster Cluster Analysis (PCCA+) algorithm that is implemented in the MSMbuilder package[23]. Notably, we found that one of the three macrostates include both pre-translocation and frayed state where the 3′-end RNA nucleotide is in frayed form but its paired DNA template nucleotide is still in the active site (i+1 site). Therefore, in order to differentiate these two biologically distinct conformations, we did another round of MSM using the MD trajectories containing only the pre-translocation and frayed states, in an effort to separate the pre-translocation states from the rest. Finally, we combined these two separately built MSMs into one 4-macrostates MSM by reassigning the newly obtained pre-translocation state from the second MSM to the first one.

Next we examine if this 4-macrostate MSM is Markovian. We first plotted the implied timescales as a function of lag time for the macrostate MSM. As shown in Supplementary

Fig. 17, we observed that the implied timescales flatten at a timescale of ~100 microseconds, consistent with what is observed for the microstate MSM (see Supplementary Fig. 9 for implied timescales for microstate models). We further performed the Chapman-Kolmogorov test to check if the macro-MSM is indeed Markovian. As this model only contains four states, we were able to apply the full version of the Chapman-Kolmogorov test to compare the time evolution of populations of all states ($P(n\tau)$) predicted by MSMs with those counted from MD trajectories, i.e. to directly examine if the following equation is satisfied: $P(n\tau) = [T(\tau)]^n P(0)$, where $T(\tau)$ is the transition probability matrix and τ is the lag time. As shown in Supplementary Fig. 18, most of the predicted state populations are in good agreement with those directly obtained from the original MD trajectories. However, we do observe some discrepancies, in particular when the system is initiated from State 3 (S3). We then concluded that the 4-state macro-MSM is not perfectly Markovian yet. Therefore, we decided to report all quantitative properties based on the microstate MSM.

Identifying the major backtracking pathway using Transition Path Theory (TPT). To find the major flux from the pre-translocation to backtracked states, we employed TPT[20,24] with the Dijkstra's algorithm[25] to identify pathways that contribute the most to the flux from the initial to final state based on our120-state MSM.

Generating 10-millisecond trajectories based on our MSM. We used the transition probability matrix of the 800-state model to produce the MSM at millisecond timescales. In particular, a random number between 0 and 1 is chosen at every step (time step is 8ns) to determine which microstate the system will transit to in the next step according to the transition probabilities out of the current microstate. In this way, we generated one 10-ms MSM simulation trajectory, from which we quantitative calculated several properties,

including the RMSD of the backbone movements, RMSF of the BH bending, and cross-correlations.

Cross-correlation calculations. To study the coupling between the motions of the DNA/RNA nucleotides and Pol II residues, we calculated the pair-wise cross-correlations. The correlation coefficient, C_{ij} between the pair i and j is defined as:

$$C_{\mathrm{ij}} = \frac{\langle \Delta \mathbf{X}_i \cdot \Delta \mathbf{X}_{\mathrm{j}} \rangle}{\left[\langle \Delta \mathbf{X}_{\mathrm{i}}^2 \rangle \langle \Delta \mathbf{X}_{\mathrm{j}}^2 \rangle \right]^{1/2}}$$

Where $\Delta \mathbf{X}_i$ is the fluctuation of the position of i with respect to its mean position. Therefore, C_{ij}=1 indicates a positively correlated motion, and C_{ij}= -1 indicates a negatively correlated motion. Center of mass of the non-hydrogen atoms for each residue/nucleotide was used in our analysis.

Supplementary note 4. The metastable macrostates identified by tICA and RMSD metrics are consistent. In order to examine if the identified metastable macrostates by using RMSD will remain the same when different metrics are applied for the MSM construction, we chose one of the models derived from the clustering based on the tICA metric (the 1000-state model under the correlation lag time of 40ns) to lump the microstates into 4 macrostates using the PCCA+ method[23]. To illustrate the structural features for each macrostate (S1-S4 states), we overlay 100 randomly selected conformations from each macrostate (see Supplementary Fig. 11). The 4 identified metastable states match very well with those observed by our previous model based on the RMSD metric (see Supplementary Fig. 12).

Supplementary note 5. Transcript cleavage assays.

Intrinsic transcript cleavage assay:

Cleavage reactions were performed by pre-incubating purified Pol II (wild type or Rpb1 T831A mutant) with the RNA:DNA scaffolds containing either rG:dC or rG:dG bp. The elongation complex was assembled with the following scaffold in a 20 mM Tris-HCl (pH = 7.5) without Mg^{2+}.

```
5'                      CGTCTGCTTATCGGTAG NTS
3'  GTAGCTCTCCTAXGCAGACGAATAGCCATC Template
5'   AUCGAGAGGAUG                         12G
              X = dG or dC
```

Intrinsic cleavage was initiated after addition of Mg^{2+}. Final concentrations for intrinsic cleavage were 20 mM Tris-HCl (pH=9), 100 nM Pol II, 25 nM scaffold, and 50 mM $MgCl_2$. The reaction was quenched in 0.5 mM EDTA at various time points and analyzed by denatured PAGE. Time points in this assay were 1 min, 5 min, 20 min, 1 hr, 3 hr, 8 hr, and 24 hr.

<u>TFIIS-aided transcript cleavage assay:</u>

Recombinant TFIIS was purified as described[26,27]. The elongation complex was assembled with the following scaffold in a 20 mM Tris-HCl (pH = 7.5) buffer without Mg^{2+}.

```
5'                        CTGCTTATCGGTAG NTS
3'  GTAGCTCTCCTAXGCAGACGAATAGCCATC Template
5'   AUCGAGAGGAUG                         12G
              X = dG or dC
```

The solution was then mixed with an equal volume of solution containing TFIIS and $MgCl_2$ in elongation buffer. Final reaction conditions were 100 nM Pol II, 25 nM scaffold, 1.5 μM TFIIS, and 5 mM $MgCl_2$. Reactions were quenched at various time points by addition of one volume of 0.5 M EDTA (pH 8.0). Products were separated by denaturing PAGE. Time points here were 5s, 15s, 30s, 1 min, 2 min, 4 min, 8 min, 15 min and 30 min.

Supplementary note 6. Backtracking for other mismatched bp systems probably also

require a frayed intermediate state. In order to check if a frayed state is also an intermediate during the backtracking process for other mismatched base pair systems, we designed three additional simulation systems with mismatched base pairs in the active site (rA:dG, rC:dT and rU:dT). If there still exist a significant fraction of MD simulations that transit toward the frayed state for these mismatched systems, it indicates that the frayed state should still be a metastable intermediate for these systems. We adopted the same TS conformations between S1 and S2 states used in the above analysis (see Fig. 3a) as the starting conformations for the new set of mutant simulations. For each of the seven conformations, we then substituted the DNA TN (dG) and the RNA 3'-end nucleotide (rG) pair to rA:dG, rC:dT and rU:dT pairs, respectively. Finally, 10-ns MD simulations were performed for each of these conformations (7 initial conformation × 3 mismatched base pairs).

As shown in Supplementary Fig. 3, for the rA:dG mismatched system, more than half of the MD conformations move towards the frayed state. For the rC:dT and rU:dT mismatched systems (Supplementary Fig. 3b and 3c, respectively), there still exist a substantial fraction of MD conformations that transit towards the frayed state, even though most of MD simulations tend to move towards the pre-translocated state. These results suggest that the frayed state is still likely to be a metastable intermediate state for backtracking in these three mismatched systems. Notably, it has been reported that a frayed rU (without backtracking) was observed in a crystal structure of the Pol II complex[28]. These experimental observations further support our results.

Supplementary References

1. Wang, D. *et al.* Structural basis of transcription: backtracked RNA polymerase II at 3.4 angstrom resolution. *Science* **324**, 1203-1206 (2009).
2. Schwede, T., Kopp, J., Guex, N. & Peitsch, M. C. SWISS-MODEL: an automated protein homology-modeling server. *Nucleic Acids Research* **31**, 3381-3385 (2003).
3. Johnson, S. J. & Beese, L. S. Structures of mismatch replication errors observed in a DNA polymerase. *Cell* **116**, 803-816 (2004).
4. Wang, D., Bushnell, D. A., Westover, K. D., Kaplan, C. D. & Kornberg, R. D. Structural basis of transcription: role of the trigger loop in substrate specificity and catalysis. *Cell* **127**, 941-954 (2006).
5. Sydow, J. F. *et al.* Structural basis of transcription: mismatch-specific fidelity mechanisms and paused RNA polymerase II with frayed RNA. *Mol. Cell* **34**, 710-721 (2009).
6. Weiss, D. R. & Levitt, M. Can morphing methods predict intermediate structures? *J. Mol. Biol.* **385**, 665-674 (2009).
7. Bowman, G. R., Huang, X. & Pande, V. S. Using generalized ensemble simulations and Markov state models to identify conformational states. *Methods* **49**, 197-201 (2009).
8. Van Der Spoel, D. *et al.* GROMACS: fast, flexible, and free. *J. Comput. Chem.* **26**, 1701-1718 (2005).
9. Berendsen, H. J. C., Postma, J. P. M., van Gunsteren, W. F. & Hermans, J. *Interaction models for water in relation to protein hydration. In B. Pullman, editor, Intermolecular forces*. 331-342 (Reidel Publishing Company, 1981).
10. Hornak, V. *et al.* Comparison of multiple Amber force fields and development of improved protein backbone parameters. *Proteins* **65**, 712-725 (2006).
11. Yildirim, I., Stern, H. A., Kennedy, S. D., Tubbs, J. D. & Turner, D. H. Reparameterization of RNA χ Torsion Parameters for the AMBER Force Field and Comparison to NMR Spectra for Cytidine and Uridine. *J. Chem. Theory Comput.* **6**, 1520-1531 (2010).
12. Zgarbová, M. *et al.* Refinement of the Cornell et al. Nucleic Acids Force Field Based on Reference Quantum Chemical Calculations of Glycosidic Torsion Profiles. *J. Chem. Theory Comput.* **7**, 2886-2902 (2011).
13. Essmann, U. *et al.* A smooth particle mesh Ewald method. *J. Chem. Phys.* **103**, 8577-8593 (1995).
14. Darden, T., York, D. & Pedersen, L. Particle mesh Ewald: An N log (N) method for Ewald sums in large systems. *J. Chem. Phys.* **98**, 10089-10092 (1993).
15. Hess, B., Bekker, H., Berendsen, H. J. C. & Fraaije, J. G. E. M. LINCS: a linear constraint solver for molecular simulations. *J. Comput. Chem.* **18**, 1463-1472 (1997).

16 Bussi, G., Donadio, D. & Parrinello, M. Canonical sampling through velocity rescaling. *J. Chem. Phys.* **126**, 014101 (2007).

17 DeLano, W. The PyMOL Molecular Graphics System on World Wide Web. *San Carlos, CA, USA: http://www.pymol. org* (2002).

18 Bowman, G. R., Voelz, V. A. & Pande, V. S. Taming the complexity of protein folding. *Curr. Opin. Struc. Biol.* **21**, 4-11 (2010).

19 Noé, F. & Fischer, S. Transition networks for modeling the kinetics of conformational change in macromolecules. *Curr. Opin. Struc. Biol.* **18**, 154-162 (2008).

20 Noé, F., Schütte, C., Vanden-Eijnden, E., Reich, L. & Weikl, T. R. Constructing the equilibrium ensemble of folding pathways from short off-equilibrium simulations. *Proc. Natl. Acad. Sci. USA* **106**, 19011-19016 (2009).

21 Swope, W. C., Pitera, J. W. & Suits, F. Describing protein folding kinetics by molecular dynamics simulations. 1. Theory. *J. Phys. Chem. B* **108**, 6571-6581 (2004).

22 Da, L.-T., Wang, D. & Huang, X. A Two-State Model for the Dynamics of the Pyrophosphate Ion Release in Bacterial RNA Polymerase. *PLoS Comput. Biol.* **9**, e1003020 (2013).

23 Deuflhard, P. & Weber, M. Robust Perron cluster analysis in conformation dynamics. *Linear Algebra And Its Applications* **398**, 161-184 (2005).

24 Weinan, E. & Vanden-Eijnden, E. Transition-path theory and path-finding algorithms for the study of rare events. *Annu. Rev. Phys.Chem.* **61**, 391-420 (2010).

25 Dijkstra, E. W. A note on two problems in connexion with graphs. *Numerische mathematik* **1**, 269-271 (1959).

26 Kellinger, M. W. *et al.* 5-formylcytosine and 5-carboxylcytosine reduce the rate and substrate specificity of RNA polymerase II transcription. *Nat. Struct. Mol. Biol.* **19**, 831-833 (2012).

27 Kellinger, M. W., Ulrich, S. b., Chong, J., Kool, E. T. & Wang, D. Dissecting chemical interactions governing RNA polymerase II transcriptional fidelity. *J. Am. Chem. Soc.* **134**, 8231-8240 (2012).

28 Sydow, J. F. *et al.* Structural basis of transcription: mismatch-specific fidelity mechanisms and paused RNA polymerase II with frayed RNA. *Mol. Cell* **34**, 710-721, (2009).
